# Supplementary material for: Effect of behavioral interventions on schistosomiasis-related knowledge, attitudes, and practices of schoolchildren in Pemba, Tanzania: A 4-year repeated cross-sectional study
Source: PLoS Negl Trop Dis. 2025 Sep 30;19(9):e0013462. doi: 10.1371/journal.pntd.0013462 (PMC12483267; doi:10.1371/journal.pntd.0013462)
Supplement: S1 Text — (PDF) [file pntd.0013462.s001.pdf]

S1 Text "Questionnaire". Questions assessed for the manuscript "Effect of behavioral interventions on schistosomiasis-related knowledge, attitudes, and practices of schoolchildren in Pemba, Tanzania: a 4-year repeated cross-sectional study" by Ndum et al. are highlighted in grey.

Questionnaire for to assess schoolchildren's knowledge, attitude and practices related to schistosomiasis

District: |__________________| Shehia: |_________________________________| **Participant ID __ __--__ __ __ __--__ __--__ __ __**

Name of school: |_________________________________|

Interviewer Name: ____ Date of interview (DD/MM/YYYY): |__|__|/|__|__|/|__|__|__|__|

**Demographics**		
	1.	Name: ____
	2.	Sex (M/F): _________
	3	Grade: _________ Grade letter: _________
	4	How old are you (in years)? ________
	5	Shehia of residency name: ____
**Schistosomiasis transmission**		
	6	Have you ever heard about schistosomiasis? ____no ___yes ____ I do not know
	7	If yes, where did you hear about schistosomiasis? ____School ____Radio ____TV ____Drug distributor ____I do not know ____ Other
	8	If other, specify__________________________
	9	What do you think is the cause of schistosomiasis? ____Blood fluke ____Worm ____I do not know …____Other

	10	If other, specify____________________________
	11	Where do you think schistosomiasis is transmitted? ____Rice field ____Toilet ____River/pond ____Trash/dump ____I do not know ____Other
	12	If other, specify____________________________
	13	During which activities do you think you get schistosomiasis? ____Playing with sand ____Playing in dirty water ____Playing in the river/pond ____Swimming in the river or pond ____Washing with river water ____Fishing from river water ____Farming with river water ____Walking barefoot ____I do not know ____Other
	14	If other, specify____________________________
	15	Do you know which animal is needed for the transmission of schistosomiasis? ____Blood fluke ____Worm ____Snail ____Bug ____I do not know ____Other
	16	If other, specify____________________________
**Schistosomiasis prevention**		
	17	Which behavior(s) can help to NOT get infected with schistosomiasis? ____Not playing in the river/pond ____Not wash in river/pond ____Not swimming in river/pond ____Use tap water/well water ____Play somewhere else than in the river/pond ____I do not know ____Other
	18	If other, specify____________________________
	19	Which behavior(s) can help to NOT transmit schistosomiasis? ____Not to urinate into river/pond ____Take treatment ____I do not know ____ Other
	20	If other, specify____________________________
**Water use**		
	21	What kind of water do you usually use for washing clothes? ____River/pond ____Tap ____Well ____I do not know ____ Other
	22	If other, specify____________________________

	23	What kind of water do you usually use for washing the dishes? ____River/pond ____Tap ____Well ____I do not know ____ Other
	24	If other, specify___________________________
	25	What kind of water do you usually use for washing your body? ____River/pond ____Tap ____Well ____I do not know ____ Other
	26	If other, specify___________________________
**River use**		
	27	Is there a river/stream or pond/lake nearby your home? ____no ___yes ____ I do not know
	28	Do you sometimes use water from the river/pond for any other purpose? ____no ___yes ____ I do not know
	29	If yes, for what purpose? ___________________________
**Schistosomiasis education at school**		
	30	Has your teacher taught you about schistosomiasis? ____no ___yes ____ I do not know
	31	When did your teacher talk last about schistosomiasis? ____This term ____Last year ____Long ago ____I do not know
	32	What kind of teaching tools was the teacher using? ____Snail board ____Flipchart ____Blood fluke picture ____No tools ___I do not know ____Other
	33	If other, specify___________________________
	33	Have you ever attended a Kichocho Day at school? ____no ___yes ____ I do not know
	35	When did you attend a Kichocho Day for the last time? ____This term ___Last year ____Long ago ____ I do not know
	36	Which activity of the Kichocho day did you like most? ____Poems ___ Games ____Drama ____ Songs ____Education ___I do not know ____Other
	37	If other, specify___________________________

**Risk factors**		
	38	Where do you typically play? ____At home (inside the house) ___ In the village (outside of the house) ____ In the forest ____ In the river/pond ____ In the bush ___Football field ___ I do not know ___ Other
	39	If other, specify___________________________
	40	Do you sometimes play in the river/pond? ____no ___yes ____ I do not know
	41	Do you cross a river/pond when you go to school? ____no ___yes ____ I do not know
	42	Do you cross a river/pond when you go to shamba? ____no ___yes ____ I do not know
	43	Do you cross a ricefield when you go to school? ____no ___yes ____ I do not know
	44	Do you cross a ricefield when you go to shamba? ____no ___yes ____ I do not know
**Laundry platforms**		
	45	Are you aware of any laundry platform installed by the kichocho team in your shehia? ____no ___yes ____ I do not know
	46	Are you using the laundry platform in your shehia? ____no ___yes ____ I do not know
	47	How often are you using the laundry platform? ____Everyday ____At least once per week ____Less than once per week ____Less than once per month ____I do not know
**Treatment**		
	48	Have you received treatment against schistosomiasis in the last 6months? ____no ___yes ____ I do not know
	49	If yes, where have you received treatment against schistosomiasis? ____MDA: at home ____MDA: in school ____T&T: at a waterbody by test-and-treat ____T&T: in school ____In a hospital ____In a health facility ____I do not know ____Other
	50	If other, specify___________________________
	51	Were you measured for your height to receive this drug? ____no ___yes ____ I do not know

	52	Were you tested for schistosomiasis before you got this drug? ____no ___yes ____ I do not know
	53	If yes, how many tablets of praziquantel did you receive? ____ 0 ____0.5 ____1 ____1.5 ____2 ____2.5 ____3 ____More than 3 ____I do not know
	54	If yes, how many tablets of praziquantel did you swallow? ____ 0 ____0.5 ____1 ____1.5 ____2 ____2.5 ____3 ____More than 3 ____I do not know
	55	Did you throw up after swallowing the drugs? ____no ___yes ____ I do not know
