# Supplementary material for: Effect of behavioral interventions on schistosomiasis-related knowledge, attitudes, and practices of schoolchildren in Pemba, Tanzania: A 4-year repeated cross-sectional study
Source: PLoS Negl Trop Dis. 2025 Sep 30;19(9):e0013462. doi: 10.1371/journal.pntd.0013462 (PMC12483267; doi:10.1371/journal.pntd.0013462)
Supplement: S1 Table — (PDF) [file pntd.0013462.s004.pdf]

S1 Table. Numbers of children responding to in questionnaire interviews about their schistosomiasis-related knowledge and attitude, in 18 schools in Pemba, Tanzania, that were either not exposed to behavior change communication (BCC), or exposed to BCC for a different frequency of intervention periods, from 2020-2024.

Table A: Children from schools that did not receive any BCC interventions

	Question	Response	Never received BCC interventions (*n* = 2084)			
			**2020 (*n* = 716)**	**2022 (*n* = 660)**	**2023 (*n* = 725)**	**2024 (*n* = 703)**
Knowledge	Where do you think schistosomiasis is transmitted?	Pond	35	0	0	0
		River	389	427	411	401
		Rice field	6	12	3	4
		Trash	9	13	2	2
		I do not know	202	177	264	206
		Other (correct/reasonable responses)	3	38	20	8
		Other (incorrect/unreasonable responses)	20	19	25	4
	During which activities do you think you get schistosomiasis?	Playing in/at/ with the river/pond	118	38	45	55
		Swimming in the river/pond	0	316	343	272
		Washing with river water	0	27	7	2
		Fishing from river water	0	3	1	0
		Farming with river water	0	0	0	0
		Walking barefoot	8	6	0	0
		Playing with sand	31	32	43	70
		Playing in dirty water	31	32	43	70’
		I do not know	463	274	231	261

		Other (correct/reasonable responses)	4	14	2	1
		Other (incorrect/unreasonable responses)	712	40	7	61
	Do you know which animal is needed for transmission of schistosomiasis?	Blood fluke	3	0	0	0
		Snail	137	65	62	42
		Worm	2	4	1	9
		I do not know	417	469	492	409
		Other (correct/reasonable responses)	2	7	1	0
		Other (incorrect/unreasonable responses)	714	57	40	89
Attitude	Which behavior(s) can help to NOT get infected with schistosomiasis?	Not playing in the river/pond	94	28	32	22
		Not washing in river/pond	23	22	8	32
		Not swimming in river/pond	186	253	256	233
		Play somewhere else than in the river/pond	6	4	2	0
		Use tap water	20	0	0	0
		Use well water	0	13	2	4
		I do not know	337	314	406	348
		Other (correct/reasonable responses)	9	18	14	14
		Other (incorrect/unreasonable responses)	97	44	19	10
	Which behavior (s) can help to NOT transmit schistosomiasis?	Not to urinate into river/pond	37	6	6	13
		Take treatment	55	28	29	91
		I do not know	571	540	624	531
		Other (correct/reasonable responses)	15	31	12	0
		Other (incorrect/unreasonable responses)	41	57	54	18

* Other (correct/reasonable responses) = Multiple listed responses with another names/Different spellings in Swahili/Spelling error in English language

* Other (incorrect/unreasonable responses) = Multiple listed responses with another names/Different spellings in Swahili/Spelling error in English language

Table B: Children from schools that received BCC interventions once

	Question	Response	Received BCC interventions once (*n* = 632)		
			**Before intervention (*n* = 183)**	**After intervention (*n* = 218)**	**After one-year gap (*n* = 231)**
Knowledge	Where do you think schistosomiasis is transmitted?	Pond	0	0	0
		River	136	200	191
		Rice field	12	22	15
		Trash	1	7	0
		I do not know	34	7	29
		Other (correct/reasonable responses)	3	15	5
		Other (incorrect/unreasonable responses)	2	6	0
	During which activities do you think you get schistosomiasis?	Playing in/at/ with the river/pond	49	69	36
		Swimming in the river/pond	0	131	162
		Washing with river water	0	32	7
		Fishing from river water	0	3	1
		Farming with river water	0	1	0
		Walking barefoot	3	13	0
		Playing with sand	0	8	0
		Playing in dirty water	15	46	10
		I do not know	112	33	44
		Other (correct/reasonable responses)	3	9	4
		Other (incorrect/unreasonable responses)	180	7	6
	Do you know which animal is needed for	Blood fluke	1	16	0
		Snail	38	79	93

	transmission of schistosomiasis?	Worm	1	19	9
		I do not know	121	61	107
		Other (correct/reasonable responses)	0	20	0
		Other (incorrect/unreasonable responses)	183	8	15
Attitude	Which behavior(s) can help to NOT get infected with schistosomiasis?	Not playing in the river/pond	34	58	10
		Not washing in river/pond	3	37	22
		Not swimming in river/pond	72	114	134
		Play somewhere else than in the river/pond	0	2	1
		Use tap water	2	0	0
		Use well water	0	22	1
		I do not know	68	47	73
		Other (correct/reasonable responses)	9	10	5
		Other (incorrect/unreasonable responses)	14	9	2
	Which behavior (s) can help to NOT transmit schistosomiasis?	Not to urinate into river/pond	15	52	19
		Take treatment	23	35	47
		I do not know	126	116	154
		Other (correct/reasonable responses)	8	12	0
		Other (incorrect/unreasonable responses)	13	11	11

* Other (correct/reasonable responses) = Multiple listed responses with another names/Different spellings in Swahili/Spelling error in English language

* Other (incorrect/unreasonable responses) = Multiple listed responses with another names/Different spellings in Swahili/Spelling error in English language

Table C: Children from schools that received BCC interventions for two consecutive periods

	Question	Response	Received BCC interventions twice with no gap (*n* = 487)		
			Before first intervention (*n* = 139)	After first intervention (*n* = 161)	After second intervention (*n* = 187)
Knowledge	Where do you think schistosomiasis is transmitted?	Pond	0	0	0
		River	114	147	175
		Rice field	3	0	41
		Trash	0	1	0
		I do not know	16	6	9
		Other (correct/reasonable responses)	5	3	0
		Other (incorrect/unreasonable responses)	4	4	1
	During which activities do you think you get schistosomiasis?	Playing in/at/ with the river/pond	30	32	54
		Swimming in the river/pond	44	13	129
		Washing with river water	1	6	24
		Fishing from river water	0	3	19
		Farming with river water	0	0	9
		Walking barefoot	1	1	1
		Playing with sand	0	0	2
		Playing in dirty water	12	8	28
		I do not know	43	18	18
		Other (correct/reasonable responses)	4	8	2
		Other (incorrect/unreasonable responses)	66	1	5
	Do you know which animal is needed for	Blood fluke	0	2	25
		Snail	37	80	118

	transmission of schistosomiasis?	Worm	3	4	12
		I do not know	75	50	52
		Other (correct/reasonable responses)	0	20	0
		Other (incorrect/unreasonable responses)	1	0	0
Attitude	Which behavior(s) can help to NOT get infected with schistosomiasis?	Not playing in the river/pond	20	29	57
		Not washing in river/pond	0	6	18
		Not swimming in river/pond	62	97	124
		Play somewhere else than in the river/pond	3	6	5
		Use tap water	0	0	0
		Use well water	2	1	4
		I do not know	45	30	36
		Other (correct/reasonable responses)	2	3	1
		Other (incorrect/unreasonable responses)	13	3	1
	Which behavior (s) can help to NOT transmit schistosomiasis?	Not to urinate into river/pond	7	18	48
		Take treatment	4	17	74
		I do not know	103	109	89
		Other (correct/reasonable responses)	16	4	2
		Other (incorrect/unreasonable responses)	9	13	10

* Other (correct/reasonable responses) = Multiple listed responses with another names/Different spellings in Swahili/Spelling error in English language

* Other (incorrect/unreasonable responses) = Multiple listed responses with another names/Different spellings in Swahili/Spelling error in English language

Table D: Children from schools that received BCC interventions twice with a 1-year gap

	Question	Response	Received BCC interventions twice with a 1-year gap (*n* = 273)			
			Before first interventions (*n* = 66)	After first interventions (*n* = 65)	After one-year gap (*n* = 71)	After second interventions (*n* = 71)
Knowledge	Where do you think schistosomiasis is transmitted?	Pond	0	0	0	0
		River	52	61	57	68
		Rice field	2	0	3	11
		Trash	0	0	0	0
		I do not know	10	1	13	2
		Other (correct/reasonable responses)	1	0	1	0
		Other (incorrect/unreasonable responses)	3	3	0	1
	During which activities do you think you get schistosomiasis?	Playing in/at/ with the river/pond	15	13	16	15
		Swimming in the river/pond	0	48	50	44
		Washing with river water	0	4	0	15
		Fishing from river water	0	5	1	7
		Farming with river water	0	0	0	4
		Walking barefoot	0	0	0	0
		Playing with sand	0	0	0	1
		Playing in dirty water	3	3	2	20’
		I do not know	43	6	18	6
		Other (correct/reasonable responses)	3	3	1	0
		Other (incorrect/unreasonable responses)	63	5	0	3
	Do you know which animal is needed for	Blood fluke	2	0	0	14
		Snail	29	40	13	47

	transmission of schistosomiasis?	Worm	0	1	3	11
		I do not know	34	19	43	12
		Other (correct/reasonable responses)	0	0	0	0
		Other (incorrect/unreasonable responses)	66	1	6	6
Attitude	Which behavior(s) can help to NOT get infected with schistosomiasis?	Not playing in the river/pond	17	27	3	27
		Not washing in river/pond	3	2	5	5
		Not swimming in river/pond	32	29	43	51
		Play somewhere else than in the river/pond	0	2	0	3
		Use tap water	0	0	0	0
		Use well water	0	1	0	2
		I do not know	18	8	23	10
		Other (correct/reasonable responses)	1	6	3	0
		Other (incorrect/unreasonable responses)	6	2	0	2
	Which behavior (s) can help to NOT transmit schistosomiasis?	Not to urinate into river/pond	8	7	2	24
		Take treatment	9	5	7	24
		I do not know	30	33	53	31
		Other (correct/reasonable responses)	5	9	3	0
		Other (incorrect/unreasonable responses)	14	12	6	3

* Other (correct/reasonable responses) = Multiple listed responses with another names/Different spellings in Swahili/Spelling error in English language

* Other (incorrect/unreasonable responses) = Multiple listed responses with another names/Different spellings in Swahili/Spelling error in English language

S1 Scoring system. System to score the responses to questions pertaining to schistosomiasis-related knowledge and attitudes of children.

Knowledge				
**S/N**	**Questions**	**Response and Scores**		
		**1**	**0.5**	**0**
1	What do you think is the cause of schistosomiasis?	Blood fluke	Worm	I do not know
				Other
2	What do you think is the cause of schistosomiasis? (other)	Slug/snail	Swimming in the river	Bacteria
		Snail	Swimming in rivers/lakes	Go to the toilet
				Disposing of dirty water
				Swimming in dirty wear
				In the pond
				Flies
				Flies and dirty water
				To eat dirty food
				Dirt
				Slug
				Disposing of dirty water
				Talking about dirty water pools
				Stagnation of water in blisters
3	Where do you think schistosomiasis is transmitted?	River/pond	River/pond and toilet	Trash/dump
		Ricefield	Ricefield and Trash/dump	Toilet
			River/pond and Trash/dump	I do not know
				Other
4		In the ponds	Pools of water	In dirty water

	Where do you think schistosomiasis is transmitted? (other)	In the rice fields	In the urine	In the sea
		In the lake and in the ponds		At home
				Bags
				Dirty places
				Bush
				Hospital
				In the desert
				At the well
				Unrelated response
5	During which activities do you think you get schistosomiasis?	Playing in the river/pond	Playing in the river/pond and walking barefoot	Playing with sand
		Swimming in the river/pond	Washing with river water and playing with sand	Playing in dirty water
		Washing with river water		Walking barefoot
		Fishing from river water		I do not know
		Farming with river water		Other
6	During which activities do you think you get schistosomiasis? (other)	Swimming in pools/ponds	Urinating in water sources (river/ponds)	Urinating in rivers
		Playing in the streams of water	Peeing in the river	Ponds
		Washing in the river	Urinating in the water	Inoculate the rain
		Bathing in the river	For urinating in the river when you have schistosomiasis	Playing in the valley
		Crossing through the river		Playing on the ropes
		Passing through rice fields		Playing cards
				Playing unsafe games
				Bleeding
				Take dewormers
				Eat mango without washing
7		Blood fluke		Bug

	Do you know which animal is needed for transmission of schistosomiasis?	Snail		Worm
				I do not know
				Other
8	Do you know which animal is needed for transmission of schistosomiasis? (other)	River snails		Malaria
		Schistoma		Eggs of insects
		Snail		Mosquito
		Slug		A female mosquito
**Attitude**				
1		Not playing in the river/pond	Use tap water/well water	I do not know
		Not wash in river/pond		Other
		Not swimming in river/pond		
		Play somewhere else than in the river/pond		
2	Which behavior(s) can help to NOT get infected with schistosomiasis?	Playing safe games	Wear shoes, use the toilet	Not protecting yourself
		Avoid playing in the sand	Do not bathe in dirty water	Not eating unwashed fruits
		Protect yourself from playing with dirty water	Do not play with dirty water	Settle at home
		Take medication for schistosomiasis	I wear shoes	Using the blood that your partner used
		Let's not bathe in rivers / swim in ponds	Do not play with dirty water	Be clean with boiled water
3			Stop messing with dirty water	Be clean
		Avoid going to rivers		Body hygiene
		Treat river		Do not play cards
		Do not bathe in dirty water		Do not play with water
		Do not wash in the river		Do not play dirty
		Do not urinate in river		Do not play with dangerous substances
		Do no swim in the river		Do not go to the valley

				Avoid playing in the sand
		Not to urinate into river/pond	Take treatment	I do not know
				Other
4	Which behavior (s) can help to NOT transmit schistosomiasis? (other)	Urinating in the toilet	Wear shoes, use the toilet	Drink clean water
		To prevent people from going to the ponds	Not swimming in pools	Drink clean water
		Do not urinate near your partner, if you go you need to clean yourself	Do not swim in rivers	Clean water bath
		Go to the hospital quickly	Do not swim in rivers/ponds	Passing urine
		Go to the doctor	Do not swim in dirty water	Cleaning the toilet after urinating
		Do not play with water	Do not swim too much	Not donating blood
		Do not go to rivers		Not playing in dirty pools
		He should not go to the rivers		Use clean water
		All schistosomiasis patients should be treated		Share things with schistosomiasis patients
		Going to the hospital and Following the doctor's advice		Use clean water
		Not swimming in pools		Be clean
				Do not hang out with other people
				Do not stay with your colleagues and do not arrest each other
				Do not stay with your partner
				Sleeping alone
				Not shaking hands
				Do not sit near the patient
