# Supplementary material for: Effect of behavioral interventions on schistosomiasis-related knowledge, attitudes, and practices of schoolchildren in Pemba, Tanzania: A 4-year repeated cross-sectional study
Source: PLoS Negl Trop Dis. 2025 Sep 30;19(9):e0013462. doi: 10.1371/journal.pntd.0013462 (PMC12483267; doi:10.1371/journal.pntd.0013462)
Supplement: S2 Table — The filled checklist is based on the STROBE Statement-Checklist of items that should be included in reports of observational studies, developed by the STROBE Initiative, https://www.strobe-statement.org/. (PDF) [file pntd.0013462.s005.pdf]

S2 Table. STROBE Statement filled checklist

This filled checklist is based on the STROBE Statement-Checklist of items that should be included in reports of observational studies, developed by the STROBE Initiative. It is licensed under a Creative Commons Attribution 4.0 International License (CC BY 4.0).

	Item No.	Recommendation	Page No.	Relevant text from manuscript
**Title and abstract**	1	(*a*) Indicate the study's design with a commonly used term in the title or the abstract	1	Effect of behavioral interventions on schistosomiasis-related knowledge, attitudes, and practices of school children in Pemba, Tanzania: a 3-year cross-sectional study.
		(*b*) Provide in the abstract an informative and balanced summary of what was done and what was found	2 & 3	**Background** Schistosomiasis is a parasitic disease that mostly affects school-age children in sub-Saharan Africa. Preventive chemotherapy is the mainstay of control. Other interventions, including behavior change communication (BCC), are recommended to reduce transmission and ultimately achieve elimination. We determined the effect of BCC interventions on schistosomiasis-related knowledge, attitudes, and practices (KAP) among children who were exposed to different BCC intervention frequencies and durations within the 4-year SchistoBreak project in Pemba, Tanzania.<br>**Methods** Annual surveys were carried out from 2020 to 2024 in 18 primary schools. Schistosomiasis-related KAP were assessed in randomly selected children attending grades 3-5. BCC interventions were implemented for one period (4 schools), two periods with no gap (3 schools), two periods with a 1-year gap (1 school), or never (10 schools). Linear and logistic mixed-models with random effect were applied to assess associations between BCC exposure categories as predictors and knowledge or attitude scores, or unsafe washing practices as the outcome variable in 2024.

				**Results:** A total of 4196 children participated in the surveys. Knowledge and attitude improved with continuous or repeated exposure to BCC interventions. In 2024, the knowledge scores were significantly higher in children who received BCC intervention once (difference: 1.2, 95% confidence interval (CI): 0.6-1.9) or twice (difference: 2.1, 95% CI: 1.4-2.7), as compared to children who never received BCC interventions. Children who were exposed to BCC interventions twice (difference: 1.2, 95% CI: 0.8-1.6) had significantly higher attitude scores in 2024. Washing practices improved regardless of whether or not children were exposed to BCC. The washing platforms installed in intervention areas were known by up to half of the children exposed to BCC interventions, but their use was considerably lower, varying between 7.5% and 43.1%.<br>**Conclusions** We demonstrated that BCC interventions were able to improve knowledge and attitudes in school children. Repeated BCC interventions might be required for sustainable long-term impact.
**Introduction**				
Background/rationale	2	Explain the scientific background and rationale for the investigation being reported	5 & 6	Schistosomiasis is a neglected tropical disease caused by parasitic blood flukes of the genus *Schistosoma*. The disease mainly occurs in tropical and subtropical countries, with the highest disease burden concentrated in sub-Saharan Africa. Individuals of all age are susceptible to *Schistosoma* infection, but school-age children are at highest risk, explained by their behavior of frequently using infested freshwater bodies. Indeed, schistosomiasis transmission occurs at freshwater bodies where children play, bathe, or fetch water for domestic chores.<br>Preventive chemotherapy with praziquantel is the global strategy to control schistosomiasis morbidity. Year after year, praziquantel is administered to millions of individuals in endemic countries, particularly to school-age children. Preventive chemotherapy

programs implemented over the past 20 years in sub-Saharan Africa have contributed to a substantial decrease in the prevalence and intensity of infection, and hence, morbidity due to schistosomiasis. In addition to preventive chemotherapy, the World Health Organization (WHO) recommends environmental interventions, improved access to water, sanitation, and hygiene (WASH), and behavior change measures to reduce transmission in endemic settings. Such an integrated intervention approach can also lead to interruption of transmission. Moreover, environmental and behavioral factors are considered essential to prevent the rebound of *Schistosoma* infections, alongside preventive chemotherapy programs and snail control measures.

Behavior change communication (BCC) is employed to deliver tailored messages concerning a particular subject to a target population, with the objective of initiating positive changes in behavior. For example, BCC can be used to improve people's health literacy. Indeed, better disease-related knowledge can change attitudes, resulting in the adoption of sustainable healthier behaviors and practices. Since *Schistosoma* infection and transmission occur at freshwater bodies, BCC intervention strategies for schistosomiasis prevention are aimed at reducing or stopping people's water contact behaviors. Hence, these strategies may include BCC for promoting the use of protective equipment (e.g., wearing boots), the adoption of ground games for children (e.g., tug of war, hopscotch, and jump rope), improving WASH, and enhanced uptake of treatment by at-risk communities.

On the Zanzibar islands, periodic preventive chemotherapy campaigns with praziquantel targeting school-age children have been implemented since the early 2000s. As a result, the prevalence of *Schistosoma haematobium* and morbidity due to urogenital schistosomiasis were substantially reduced. In 2010, efforts to eliminate urogenital schistosomiasis as a public health

				problem and to interrupt transmission were intensified. In addition to preventive chemotherapy provided in schools and communities across the islands, snail control and BCC measures were implemented in selected schools and communities as part of specific research projects. In the SchistoBreak project, conducted from 2020 to 2024, *S. haematobium* hotspot areas in the North of Pemba received a combination of preventive chemotherapy, snail control, and BCC interventions, while low-prevalence areas were subjected to a surveillance-response approach.
Objectives	3	State specific objectives, including any prespecified hypotheses	6	The purpose of this study was to determine the effect of BCC interventions on schistosomiasis-related knowledge, attitudes, and practices (KAP) of schoolchildren that were exposed to different frequencies and durations of BCC interventions within the 4-year SchistoBreak project. Moreover, we assessed a potential association of the BCC exposure frequency with KAP or *S. haematobium* infection, as determined in the final survey of the SchistoBreak project in 2024.
**Methods**				
Study design	4	Present key elements of study design early in the paper	8	The SchistoBreak project followed a longitudinal design with four annual school-based and household-based surveys that used a cross-sectional sampling approach and three annual intervention periods.
Setting	5	Describe the setting, locations, and relevant dates, including periods of recruitment, exposure, follow-up, and data collection	8	The SchistoBreak project was conducted on Pemba Island from 2020 to 2024.
			8	The SchistoBreak project area consisted of 20 shehias in Wete and Micheweni districts.
			6	In the SchistoBreak project, conducted from 2020 to 2024, *S. haematobium* hotspot areas in the North of Pemba received a combination of preventive chemotherapy, snail control, and BCC interventions, while low-prevalence areas were subjected to a surveillance-response approach.

			13	To assess a potential association of the number of intervention period categories (never, once, twice with no gap, and twice with a 1-year gap) with knowledge or attitudes of children, respectively, as categorical variables, a linear mixed-model with random effects was employed, adjusting for age and sex, and including the school as cluster variable.
			13	Subsequently, Jenks natural break was employed to classify the respective cumulative scores across all four surveys into distinct categories by minimizing variance within each category and maximizing variance between categories.
Participants	6	(*a*) *Cohort study*—Give the eligibility criteria, and the sources and methods of selection of participants. Describe methods of follow-up		
		Case-control study—Give the eligibility criteria, and the sources and methods of case ascertainment and control selection. Give the rationale for the choice of cases and controls		
		Cross-sectional study—Give the eligibility criteria, and the sources and methods of selection of participants	10 & 11	Children from nursery and grades 1-6 were eligible to participate in the parasitologic surveys, while children attending grades 3-5 were additionally eligible to participate in the KAP surveys. If a school had more than one class per grade, a computer-generated randomization list was employed to select either class A, B, C, or D, respectively. In each selected class per grade, 25 children were randomly selected for participation, using the following approach: all children in a class queued up, stratified by sex, and every third child in each line was systematically selected to be included in the study until 25 children were recruited.
		(*b*) *Cohort study*—For matched studies, give matching criteria and number of exposed and unexposed		

		Case-control study—For matched studies, give matching criteria and the number of controls per case		
Variables	7	Clearly define all outcomes, exposures, predictors, potential confounders, and effect modifiers. Give diagnostic criteria, if applicable	13<br><br>11<br><br><br><br><br><br><br>14<br><br><br><br><br><br><br>14<br><br><br><br><br><br><br>14	*S. haematobium* infection, knowledge, attitude and practices.<br><br>The questionnaire inquired about demographic data (e.g., age, sex, and shehia of residence of the child) and children's KAP regarding schistosomiasis transmission and prevention (e.g., knowledge of the lifecycle of *S. haematobium*, measures to prevent infection and avoid the spread of *S. haematobium*, and attitude toward schistosomiasis prevention and water contact practices).<br><br>To assess a potential association of the number of intervention period categories (never, once, twice with no gap, and twice with a 1-year gap) with knowledge or attitudes of children, respectively, as categorical variables, a linear mixed-model with random effects was employed, adjusting for age and sex, and including the school as cluster variable.<br><br>To assess a potential association of the number of intervention period categories (never, once, twice with no gap, and twice with a 1-year gap) with knowledge or attitudes of children, respectively, as categorical variables, a linear mixed-model with random effects was employed, adjusting for age and sex, and including the school as cluster variable.<br><br>Participants were considered as *S. haematobium*-positive if at least one *S. haematobium* egg was detected in 10 ml of their urine.
Data sources/ measurement	8*	For each variable of interest, give sources of data and details of methods of assessment (measurement). Describe comparability of assessment methods if there is more than one group	12<br><br><br>12	In the laboratory, urine samples were subjected to a filtration method.<br><br><br>The presence and number of *S. haematobium* eggs were recorded on paper case report forms.

			11	Children from grades 3-5 were also invited to participate in a questionnaire interview about schistosomiasis-related KAP.
			11	The questionnaire inquired about demographic data (e.g., age, sex, and shehia of residence of the child) and children's KAP regarding schistosomiasis transmission and prevention (e.g., knowledge of the lifecycle of *S. haematobium*, measures to prevent infection and avoid the spread of *S. haematobium*, and attitude toward schistosomiasis prevention and water contact practices).
Bias	9	Describe any efforts to address potential sources of bias	14 & 15	Of note, the categories for three schools that were exposed to BCC interventions twice with no gap and for the one school that was exposed to BCC interventions twice with a 1-year gap were combined to gain enough clusters for the analyses.
Study size	10	Explain how the study size was arrived at	11	In each selected class per grade, 25 children were randomly selected for participation, using the following approach: all children in a class queued up, stratified by sex, and every third child in each line was systematically selected to be included in the study until 25 children were recruited. Details of the sample size calculations are provided in the published study protocol.

Continued on next page

Quantitative variables	11	Explain how quantitative variables were handled in the analyses. If applicable, describe which groupings were chosen and why	13	Participants' sociodemographic data (i.e., sex, age, and school grade) were analyzed descriptively and presented as frequencies and percentages. Data from the KAP questionnaires were analyzed as follows: each of the three knowledge and two attitude multiple-choice questions were scored as incorrect (0 points), partially correct (0.5 points), and correct (1 point) with a cumulative highest possible score of 13 for knowledge and 9 for attitude, respectively, per annual survey. Subsequently, Jenks natural break was employed to classify the respective cumulative scores across all four surveys into distinct categories by minimizing variance within each category and maximizing variance between categories. Knowledge about schistosomiasis was classified into four categories: no knowledge (0 points), poor (1.0-1.5 points), moderate (1.5-2.5 points), and good (2.5-8.0 points) knowledge. Attitude was classified into three categories: poor (0-0.5 points), moderate (0.5-1.5 points), and good (1.5-5.5 points).
			13	Practices for schistosomiasis prevention were analyzed descriptively from three questions about the main source of water children used for washing their bodies, dishes or clothes, respectively. Furthermore, data from the first and final surveys conducted at the start and end of the SchistoBreak project in 2020 and 2024, respectively, were used to generate box plots, which illustrate the distribution of knowledge and attitude scores, respectively, of children who were exposed to BCC for different intervention periods.
			14	Participants were considered as *S. haematobium*-positive if at least one *S. haematobium* egg was detected in 10 ml of their urine.
Statistical methods	12	(*a*) Describe all statistical methods, including those used to control for confounding	14	To assess a potential association of the number of intervention period categories (never, once, twice with no gap, and twice with a 1-year gap) with knowledge or attitudes of children, respectively, as categorical variables, a linear mixed-model with random effects was employed, adjusting for age and sex, and including the school as cluster variable. Of note, the categories for three schools that were exposed to BCC interventions twice with no gap and for the one school that was exposed to BCC interventions twice with a 1-year gap were combined to gain enough clusters for the analyses. The results were

			presented as estimated difference and 95% confidence intervals (CIs) using forest plots. Statistical significance was considered when the 95% CI of the difference did not include 0.<br>To assess a potential association of the number of intervention period categories (never, once, twice with no gap, and twice with a 1-year gap) with washing practices or *S. haematobium* infection, respectively, as binary variables, a logistic mixed-model with random effects was used, adjusting for age and sex, and including school as cluster variable. Washing practices were stratified into safe (if a child used either tap or well water for all washing practices) or unsafe (if a child used river/pond water for any washing practice). Participants were considered as *S. haematobium*-positive if at least one *S. haematobium* egg was detected in 10 ml of their urine. Of note, the categories for three schools that were exposed to BCC interventions twice with no gap and for the one school that was exposed to BCC interventions twice with a 1-year gap were combined to gain enough clusters for the analyses. The results were presented as odds ratio (OR) including 95% CIs using forest plots. Statistical significance was considered when the 95% CI did not include 1 for OR.
	(*b*) Describe any methods used to examine subgroups and interactions	14	To assess a potential association of the number of intervention period categories (never, once, twice with no gap, and twice with a 1-year gap) with knowledge or attitudes of children, respectively, as categorical variables, a linear mixed-model with random effects was employed, adjusting for age and sex, and including the school as cluster variable. Of note, the categories for three schools that were exposed to BCC interventions twice with no gap and for the one school that was exposed to BCC interventions twice with a 1-year gap were combined to gain enough clusters for the analyses. The results were presented as estimated difference and 95% confidence intervals (CIs) using forest plots. Statistical significance was considered when the 95% CI of the difference did not include 0.<br>To assess a potential association of the number of intervention period categories (never, once, twice with no gap, and twice with a 1-year gap) with washing practices or *S. haematobium* infection, respectively, as binary variables, a logistic mixed-model with random effects was used, adjusting for age and sex, and including school as cluster variable. Washing practices were

			stratified into safe (if a child used either tap or well water for all washing practices) or unsafe (if a child used river/pond water for any washing practice). Participants were considered as *S. haematobium*-positive if at least one *S. haematobium* egg was detected in 10 ml of their urine. Of note, the categories for three schools that were exposed to BCC interventions twice with no gap and for the one school that was exposed to BCC interventions twice with a 1-year gap were combined to gain enough clusters for the analyses. The results were presented as odds ratio (OR) including 95% CIs using forest plots. Statistical significance was considered when the 95% CI did not include 1 for OR.
	(*c*) Explain how missing data were addressed	17 & Fig .2<br><br>14	**Fig 2.** Exposure categories of schools to behavior change communication (BCC) interventions and participation in surveys from 2020-2024. Schools participation in behavior change communication (BCC) intervention periods and participation of children in questionnaire interviews about their schistosomiasis-related knowledge, attitude, and practices (KAP), and urine examinations for determining *Schistosoma haematobium* infection in Pemba, Tanzania, from 2020-2024.<br><br>To ensure consistent handling of missing data (coded in R as "NA" [not available]) across the variables used in the models, conditional logic was applied. Hence, by default in R, the statistical analysis excluded all observations in variables containing missing data.
	(*d*) *Cohort study*—If applicable, explain how loss to follow-up was addressed<br>*Case-control study*—If applicable, explain how matching of cases and controls was addressed<br>*Cross-sectional study*—If applicable, describe analytical methods taking account of sampling strategy	14	To assess a potential association of the number of intervention period categories (never, once, twice with no gap, and twice with a 1-year gap) with knowledge or attitudes of children, respectively, as categorical variables, a linear mixed-model with random effects was employed, adjusting for age and sex, and including the school as cluster variable. Of note, the categories for three schools that were exposed to BCC interventions twice with no gap and

				for the one school that was exposed to BCC interventions twice with a 1-year gap were combined to gain enough clusters for the analyses. The results were presented as estimated difference and 95% confidence intervals (CIs) using forest plots. Statistical significance was considered when the 95% CI of the difference did not include 0.<br>To assess a potential association of the number of intervention period categories (never, once, twice with no gap, and twice with a 1-year gap) with washing practices or *S. haematobium* infection, respectively, as binary variables, a logistic mixed-model with random effects was used, adjusting for age and sex, and including school as cluster variable. Washing practices were stratified into safe (if a child used either tap or well water for all washing practices) or unsafe (if a child used river/pond water for any washing practice). Participants were considered as *S. haematobium*-positive if at least one *S. haematobium* egg was detected in 10 ml of their urine. Of note, the categories for three schools that were exposed to BCC interventions twice with no gap and for the one school that was exposed to BCC interventions twice with a 1-year gap were combined to gain enough clusters for the analyses. The results were presented as odds ratio (OR) including 95% CIs using forest plots. Statistical significance was considered when the 95% CI did not include 1 for OR.
		(*e*) Describe any sensitivity analyses		
**Results**				
Participants	13*	(a) Report numbers of individuals at each stage of study—eg numbers potentially eligible, examined for eligibility, confirmed eligible, included in the study, completing follow-up, and analysed	15	Over the whole SchistoBreak project, a total of 4626 children attending grades 3-5 in 18 schools were registered to participate in the study. Among them, 2852 children visited 10 schools that never received BCC interventions, 864 were from four schools that received BCC in one period, 632 were from three schools that received BCC interventions for two periods with no gap, and 278 were from the remaining school that received BCC interventions twice with a 1-year gap (Fig 2). Among the registered children, 79 did not participate in the KAP questionnaires.<br>From the 4547 students who participated in the KAP questionnaire surveys, the analyses included only those who visited a school that never received BCC interventions, or who participated in the surveys directly preceding or

			following a BCC intervention period. Hence, the KAP data from 2804 children who never received BCC, 632 children who received BCC once, 487 children who received BCC twice with no gap, and 273 children who received BCC twice with a 1-year gap were analyzed. Among those groups, 10, 8, 2, and 1 children, respectively, lacked urine filtration data.<br>In the 10 schools that never received the BCC interventions, 716 children participated in the KAP questionnaire survey in 2020, 660 in 2022, 725 in 2023, and 703 in 2024. In the four schools that received the BCC interventions for one period, 183 children were interviewed before and 218 children after the intervention. Additionally, 231 children were interviewed after the BCC interventions had stopped for 1 year. In the three schools that received the BCC interventions for two periods without a gap, 139 children were interviewed before the first, 161 children after the first and before the second intervention period, and 187 children after the second intervention period. In the school that received the interventions twice with a 1-year gap, 66 children participated before the first BCC intervention, 65 after the first intervention, 71 after the 1-year gap, and 71 after the second intervention period.<br>Of the 4196 schoolchildren who were included in the statistical analyses, 2148 (51.2%) were females (Table 1). Participants included 1414 (33.7%) children from grade 3, 1414 (33.7%) children from grade 4, and 1368 (32.6%) children from grade 5.
	(b) Give reasons for non-participation at each stage	15	Over the whole SchistoBreak project, a total of 4626 children attending grades 3-5 in 18 schools were registered to participate in the study. Among them, 2852 children visited 10 schools that never received BCC interventions, 864 were from four schools that received BCC in one period, 632 were from three schools that received BCC interventions for two periods with no gap, and 278 were from the remaining school that received BCC interventions twice with a 1-year gap (Fig 2). Among the registered children, 79 did not participate in the KAP questionnaires.
	(c) Consider use of a flow diagram	17	**Fig 2.** Exposure categories of schools to behavior change communication (BCC) interventions and participation in surveys from 2020-2024. Schools participation in behavior change communication (BCC) intervention periods and participation of children in questionnaire interviews about their schistosomiasis-related knowledge, attitude, and practices (KAP), and urine

				examinations for determining *Schistosoma haematobium* infection in Pemba, Tanzania, from 2020-2024.
Descriptive data	14*	(a) Give characteristics of study participants (eg demographic, clinical, social) and information on exposures and potential confounders	16, 18 & Table 1	Of the 4196 schoolchildren who were included in the statistical analyses, 2148 (51.2%) were females (Table 1). Participants included 1414 (33.7%) children from grade 3, 1414 (33.7%) children from grade 4, and 1368 (32.6%) children from grade 5. Their interquartile age ranged from 10 to 12 years, with a median age of 11 years. Demographic details for each intervention exposure category group are summarized in Table 1.
		(b) Indicate number of participants with missing data for each variable of interest	15, 16 & Fig 2.	Over the whole SchistoBreak project, a total of 4626 children attending grades 3-5 in 18 schools were registered to participate in the study. Among them, 2852 children visited 10 schools that never received BCC interventions, 864 were from four schools that received BCC in one period, 632 were from three schools that received BCC interventions for two periods with no gap, and 278 were from the remaining school that received BCC interventions twice with a 1-year gap (Fig 2). Among the registered children, 79 did not participate in the KAP questionnaires.<br>From the 4547 students who participated in the KAP questionnaire surveys, the analyses included only those who visited a school that never received BCC interventions, or who participated in the surveys directly preceding or following a BCC intervention period. Hence, the KAP data from 2804 children who never received BCC, 632 children who received BCC once, 487 children who received BCC twice with no gap, and 273 children who received BCC twice with a 1-year gap were analyzed. Among those groups, 10, 8, 2, and 1 children, respectively, lacked urine filtration data.
			17	**Fig 2.** Exposure categories of schools to behavior change communication (BCC) interventions and participation in surveys from 2020-2024. Schools participation in behavior change communication (BCC) intervention periods and participation of children in questionnaire interviews about their schistosomiasis-related knowledge, attitude, and practices (KAP), and urine examinations for determining *Schistosoma haematobium* infection in Pemba, Tanzania, from 2020-2024.

		(c) *Cohort study*—Summarise follow-up time (eg, average and total amount)		
Outcome data	15*	*Cohort study*—Report numbers of outcome events or summary measures over time		
		Case-control study—Report numbers in each exposure category, or summary measures of exposure		
		Cross-sectional study—Report numbers of outcome events or summary measures	15 & 16	In the 10 schools that never received the BCC interventions, 716 children participated in the KAP questionnaire survey in 2020, 660 in 2022, 725 in 2023, and 703 in 2024. In the four schools that received the BCC interventions for one period, 183 children were interviewed before and 218 children after the intervention. Additionally, 231 children were interviewed after the BCC interventions had stopped for 1 year. In the three schools that received the BCC interventions for two periods without a gap, 139 children were interviewed before the first, 161 children after the first and before the second intervention period, and 187 children after the second intervention period. In the school that received the interventions twice with a 1-year gap, 66 children participated before the first BCC intervention, 65 after the first intervention, 71 after the 1-year gap, and 71 after the second intervention period.
Main results	16	(*a*) Give unadjusted estimates and, if applicable, confounder-adjusted estimates and their precision (eg, 95% confidence interval). Make clear which confounders were adjusted for and why they were included	14	To assess a potential association of the number of intervention period categories (never, once, twice with no gap, and twice with a 1-year gap) with knowledge or attitudes of children, respectively, as categorical variables, a linear mixed-model with random effects was employed, adjusting for age and sex, and including the school as cluster variable.<br>To assess a potential association of the number of intervention period categories (never, once, twice with no gap, and twice with a 1-year gap) with washing practices or *S. haematobium* infection, respectively, as binary variables, a logistic mixed-model with random effects was used, adjusting for age and sex, and including school as cluster variable.

	(*b*) Report category boundaries when continuous variables were categorized	13	Data from the KAP questionnaires were analyzed as follows: each of the three knowledge and two attitude multiple-choice questions were scored as incorrect (0 points), partially correct (0.5 points), and correct (1 point) with a cumulative highest possible score of 13 for knowledge and 9 for attitude, respectively, per annual survey. Subsequently, Jenks natural break was employed to classify the respective cumulative scores across all four surveys into distinct categories by minimizing variance within each category and maximizing variance between categories. Knowledge about schistosomiasis was classified into four categories: no knowledge (0 points), poor (1.0-1.5 points), moderate (1.5-2.5 points), and good (2.5-8.0 points) knowledge. Attitude was classified into three categories: poor (0-0.5 points), moderate (0.5-1.5 points), and good (1.5-5.5 points).
	(*c*) If relevant, consider translating estimates of relative risk into absolute risk for a meaningful time period		

Continued on next page

Other analyses	17	Report other analyses done—eg analyses of subgroups and interactions, and sensitivity analyses	27 & Fig 6	**Fig 6.** Distribution of knowledge scores (A) and attitude scores (B) of children from 18 schools in Pemba, Tanzania, who were either not exposed to behavior change communication (BCC) (n = 10 schools), or exposed to BCC for one intervention period (n = 4 schools), for two intervention periods without a gap (n = 3 schools), or for two intervention periods with a 1-year gap (n = 1 school) in the final survey of the SchistoBreak project in 2024.
			29, 30 & Fig 7	**Fig 7**. Association of behavior change communication (BCC) exposure frequency with knowledge score, attitude score, practices and *S. haematobium* infection. Association of behavior change communication (BCC) exposure frequency using a linear mixed-model for children's knowledge (A) and attitude (B) and using a logistic mixed-model for children's washing practices (C) and *S. haematobium* infection (D), as assessed in the final survey of the SchistoBreak project in Pemba, Tanzania, in 2024.
**Discussion**				
Key results	18	Summarise key results with reference to study objectives	30	Our study showed that knowledge increased with repeated BCC intervention exposure. Moreover, children who received BCC interventions once or twice had considerably better schistosomiasis-related knowledge than those who never received BCC interventions at the end of the study. Good knowledge increased with repeated exposure. However, once BCC interventions were terminated or interrupted for a certain period, the percentage of children with good knowledge decreased.
			30 & 31	Interestingly, children from BCC intervention schools had more schistosomiasis-related knowledge preceding the interventions than children from non-intervention schools. Since the former schools were located in hotspot areas, where schistosomiasis has been a public health problem for a long time, children were probably more aware of the disease and its transmission than their counterparts from low-prevalence areas. Many children who never received BCC interventions had no knowledge about schistosomiasis and the existing levels of knowledge remained relatively stable over time. These findings illustrate that knowledge acquired through

				BCC interventions is improved and accumulated over time and that long-term and repeated exposure to BCC is needed for a lasting effect.
			31	The BCC interventions applied in our study also had an obvious impact on the schistosomiasis-related attitude of children. Indeed, children who received BCC interventions once or twice had significantly higher attitude scores than unexposed children in the final survey. Good and moderate attitude, which was reflected in knowledge about behaviors that can help not to get infected with or transmit *S. haematobium* also increased over time with continuous or repeated implementation of BCC interventions. The attitudes that were assessed in our study were about behaviors that can prevent *S. haematobium* infection and transmission, including not urinating, playing, washing, or swimming in the river, but instead using clean water for washing, playing elsewhere, and taking treatment.
			32	A substantial decline in the use of open freshwater bodies for washing clothes or dishes and bathing was found in children visiting schools of all investigated BCC exposure categories. Additionally, a profound decrease was determined after the first intervention period and children who were exposed to BCC measures for two periods hardly reported the use of any natural open water bodies after the second intervention. However, a decrease was also detected in children from schools that were never exposed to BCC interventions, except for the final survey of our study.
Limitations	19	Discuss limitations of the study, taking into account sources of potential bias or imprecision. Discuss both direction and magnitude of any potential bias	33	This study has specific strengths and limitations. We consider as strength and novelty that a new BCC intervention approach was investigated in a 4-year study, allowing to assess an effect on schistosomiasis-related KAP across different periods of implementation. Important evidence was generated that documented the gain or loss of KAP with extended periods of intervention or when interventions are paused or stopped, respectively. Limitations of the study include the discrepancy in sample sizes in each intervention period, especially the very small number of participants in the category that received BCC intervention twice with a 1-year gap, which was not ideal for comparison. They also include the already existing schistosomiasis-related knowledge and attitude that children who attended hotspot schools had prior

				to the onset of our BCC interventions. Of note, after adjusting for the respective mean knowledge score and attitude score per school at baseline, the interpretation of our results in the final survey did not change. The random sampling approach used in the cross-sectional annual surveys did not allow the possibility to account for the change in knowledge and attitude scores for individual study participants between the surveys in 2020 and 2024. Hence, future studies may consider larger sample sizes in all comparison groups and assessing the effect of BCC in a "naïve" population.
Interpretation	20	Give a cautious overall interpretation of results considering objectives, limitations, multiplicity of analyses, results from similar studies, and other relevant evidence	33 & 34	In our study, knowledge and attitude about the life cycle of *S. haematobium*, places of transmission, and where and during what activities the parasite is transmitted increased with continuous or repeated exposure to BCC interventions, but was partially lost after 1-year gap in exposure or when interventions ceased altogether. Reported washing practices like avoiding natural open freshwater bodies improved in children exposed and unexposed to BCC interventions. The few washing platforms installed in hotspot shehias were partially known but not much used by the study participants. For a maximum and lasting effect on KAP, BCC interventions should be applied over a long time and consolidated by improved access to WASH infrastructure in schools and communities. Only when people know and are reminded repeatedly about the transmission route and consequences of schistosomiasis and when they have access to adequate alternatives, they may change their behavior to prevent infection and transmission, which will ultimately support elimination.
Generalisability	21	Discuss the generalisability (external validity) of the study results	31	A positive impact of long-term BCC was also observed in Mozambique, where community members showed an improvement in knowledge about schistosomiasis after receiving a 1-year community dialogue intervention. Moreover, studies from Brazil and a prior study in Zanzibar, where schistosomiasis-related BCC interventions were implemented for 2 and 4 years, respectively, showed that long-term exposure can have beneficial effects in creating awareness for preventive behaviors among children. Similar findings were reported in Ethiopia, where BCC interventions had a significant impact on improving knowledge and practices for other neglected tropical diseases, like soil-transmitted helminthiasis and onchocerciasis.

			31 & 32	As discussed elsewhere and revealed by our study, learning and understanding need repeated exposure and take time, as does a change in behavior. Moreover, behavior change does not only need time but also alternatives to the use of open freshwater bodies (e.g., access to improved WASH).
			32	Hence, while children who were exposed to BCC interventions had likely adopted safer washing practices in line with what they learned, it may also be that the general water infrastructure in the North of Pemba was improved, as reported from other areas in Zanzibar.
			34	For a maximum and lasting effect on KAP, BCC interventions should be applied over a long time and consolidated by improved access to WASH infrastructure in schools and communities. Only when people know and are reminded repeatedly about the transmission route and consequences of schistosomiasis and when they have access to adequate alternatives, they may change their behavior to prevent infection and transmission, which will ultimately support elimination.
**Other information**				
Funding	22	Give the source of funding and the role of the funders for the present study and, if applicable, for the original study on which the present article is based	35	This study obtained funding from the Swiss National Science Foundation (SNSF) via a PRIMA grant to Stefanie Knopp (PR00P3_179753). Naomi C. Ndum is supported by a personal stipend from the Swiss Government Excellence Scholarships (ESKAS) program and the Swiss Tropical and Public Health Institute (Swiss TPH). The funding sources had no role in the design and execution of this study, analyses and interpretation of the data, or decision to submit the manuscript for publication.

*Give information separately for cases and controls in case-control studies and, if applicable, for exposed and unexposed groups in cohort and cross-sectional studies.

Note: An Explanation and Elaboration article discusses each checklist item and gives methodological background and published examples of transparent reporting. The STROBE checklist is best used in conjunction with this article (freely available on the Web sites of PLoS Medicine at http://www.plosmedicine.org/, Annals of Internal Medicine at http://www.annals.org/, and Epidemiology at http://www.epidem.com/). Information on the STROBE Initiative is available at www.strobe-statement.org.
