# Supplementary material for: Effect of behavioral interventions on schistosomiasis-related knowledge, attitudes, and practices of schoolchildren in Pemba, Tanzania: A 4-year repeated cross-sectional study
Source: PLoS Negl Trop Dis. 2025 Sep 30;19(9):e0013462. doi: 10.1371/journal.pntd.0013462 (PMC12483267; doi:10.1371/journal.pntd.0013462)
Supplement: Attachment — Submitted filename: MI_PNTD-D-25-00558_reviewer_.pdf [file pntd.0013462.s006.pdf]

PLOS Neglected Tropical Diseases

Effect of behavioral interventions on schistosomiasis-related knowledge, attitudes, and practices of school children in Pemba, Tanzania: a 4-year study

--Manuscript Draft--

**Manuscript Number:**	PNTD-D-25-00558
**Full Title:**	Effect of behavioral interventions on schistosomiasis-related knowledge, attitudes, and practices of school children in Pemba, Tanzania: a 4-year study
**Short Title:**	Behavior change communication for schistosomiasis elimination
**Article Type:**	Research Article
**Keywords:**	Attitude; Behavior change communication; control; Elimination; intervention; Knowledge; Practices; schistosomiasis; Tanzania
**Abstract:**	Background Schistosomiasis is a parasitic disease that mostly affects school-age children in sub-Saharan Africa. Preventive chemotherapy is the mainstay of control. Other interventions, including behavior change communication (BCC), are recommended to reduce transmission and ultimately achieve elimination. We determined the effect of BCC interventions on schistosomiasis-related knowledge, attitudes, and practices (KAP) among children who were exposed to different BCC intervention frequencies and durations within the 4-year SchistoBreak project in Pemba, Tanzania.<br><br>Methods Annual surveys were carried out from 2020 to 2024 in 18 primary schools. Schistosomiasis-related KAP were assessed in randomly selected children attending grades 3-5. BCC interventions were implemented for one period (4 schools), two periods with no gap (3 schools), two periods with a 1-year gap (1 school), or never (10 schools). Linear and logistic mixed-models with random effect were applied to assess associations between BCC exposure categories as predictors and knowledge or attitude scores, or unsafe washing practices as the outcome variable in 2024.<br><br>Results: A total of 4196 children participated in the surveys. Knowledge and attitude improved with continuous or repeated exposure to BCC interventions. In 2024, the knowledge scores were significantly higher in children who received BCC intervention once (difference: 1.2, 95% confidence interval (CI): 0.6-1.9) or twice (difference: 2.1, 95% CI: 1.4-2.7), as compared to children who never received BCC interventions. Children who were exposed to BCC interventions twice (difference: 1.2, 95% CI: 0.8-1.6) had significantly higher attitude scores in 2024. Washing practices improved regardless of whether or not children were exposed to BCC. The washing platforms installed in intervention areas were known by up to half of the children exposed to BCC interventions, but their use was considerably lower, varying between 7.5% and 43.1%.<br><br>Conclusions We demonstrated that BCC interventions were able to improve knowledge and attitudes in school children. Repeated BCC interventions might be required for sustainable long-term impact.<br><br>Trial registration ISRCTN, ISRCTN91431493. Registered 11 February. 2020, https://www.isrctn.com/ISRCTN91431493
**Additional Information:**	
**Question**	**Response**
**Government Employee**<br><br>Are you or any of the contributing authors an employee of the United States government?	No - No authors are employees of the U.S. government.

Powered by Editorial Manager® and ProduXion Manager® from Aries Systems Corporation

Manuscripts authored by one or more US Government employees are not copyrighted, but are licensed under a CC0 Public Domain Dedication, which allows unlimited distribution and reuse of the article for any lawful purpose. This is a legal requirement for US Government employees.<br><br>This will be typeset if the manuscript is accepted for publication.	
**Financial Disclosure**<br><br>Enter a financial disclosure statement that describes the sources of funding for the work included in this submission. Review the submission guidelines for detailed requirements. View published research articles from *PLOS Neglected Tropical Diseases* for specific examples.<br><br>This statement is required for submission and **will appear in the published article** if the submission is accepted. Please make sure it is accurate.<br><br>**Funded studies**<br>Enter a statement with the following details:<br>• Initials of the authors who received each award<br>• Grant numbers awarded to each author<br>• The full name of each funder<br>• URL of each funder website<br>• Did the sponsors or funders play any role in the study design, data collection and analysis, decision to publish, or preparation of the manuscript?<br><br>Did you receive funding for this work?	Yes
Please add funding details.<br>as follow-up to "**Financial Disclosure**<br><br>Enter a financial disclosure statement that describes the sources of funding for the work included in this submission. Review the submission guidelines for detailed requirements. View published research	This study obtained funding from the Swiss National Science Foundation (SNSF) via a PRIMA grant to Stefanie Knopp (PR00P3_179753). Naomi C. Ndum is supported by a personal stipend from the Swiss Government Excellence Scholarships (ESKAS) program and the Swiss Tropical and Public Health Institute (Swiss TPH). The funders had no role in study design, data collection and analysis, decision to publish, or preparation of the manuscript.

articles from *PLOS Neglected Tropical Diseases* for specific examples.<br><br>This statement is required for submission and **will appear in the published article** if the submission is accepted. Please make sure it is accurate.<br><br>**Funded studies**<br>Enter a statement with the following details:<br>• Initials of the authors who received each award<br>• Grant numbers awarded to each author<br>• The full name of each funder<br>• URL of each funder website<br>• Did the sponsors or funders play any role in the study design, data collection and analysis, decision to publish, or preparation of the manuscript?<br><br>Did you receive funding for this work?"	
Please select the country of your main research funder (please select carefully as in some cases this is used in fee calculation).<br>as follow-up to "**Financial Disclosure**<br><br>Enter a financial disclosure statement that describes the sources of funding for the work included in this submission. Review the submission guidelines for detailed requirements. View published research articles from *PLOS Neglected Tropical Diseases* for specific examples.<br><br>This statement is required for submission and **will appear in the published article** if the submission is accepted. Please make sure it is accurate.	SWITZERLAND - CH

**Funded studies**<br>Enter a statement with the following details:<br>• Initials of the authors who received each award<br>• Grant numbers awarded to each author<br>• The full name of each funder<br>• URL of each funder website<br>• Did the sponsors or funders play any role in the study design, data collection and analysis, decision to publish, or preparation of the manuscript?<br><br>Did you receive funding for this work?"	
**Competing Interests**<br><br>On behalf of all authors, disclose any competing interests that could be perceived to bias this work.<br><br>This statement will be typeset if the manuscript is accepted for publication.<br><br>Review the instructions link below and PLOS NTDs' competing interests policy to determine what information must be disclosed at submission.	The authors have declared that no competing interests exist.
**Data Availability**<br><br>Provide a **Data Availability Statement** in the box below. This statement should detail where the data used in this submission can be accessed. This statement will be typeset if the manuscript is accepted for publication.<br><br>Before publication, authors are required to make all data underlying their findings fully available, without restriction. Review our PLOS Data Policy page for detailed information on this policy. Instructions for writing your Data Availability statement can be accessed via the Instructions link below.	All relevant data are within the manuscript and its Supporting Information files.

Powered by Editorial Manager® and ProduXion Manager® from Aries Systems Corporation

Manuscript

Click here to access/download;Manuscript;Ndum_School KAP2_FINAL_16.04.2025.docx

Effect of behavioral interventions on schistosomiasis-related knowledge, attitudes, and practices of school children in Pemba, Tanzania: a 4-year study

Naomi C. Ndum[1,2], Lydia Trippler[1,2,3], Ulfat A. Mohammed[4], Mohammed N. Ali[4], Jan Hattendorf[1,2], Shaali M. Ame[5], Fatma Kabole[5], Jürg Utzinger[1,2], Said M. Ali[4], Stefanie Knopp[1,2*]

1 Swiss Tropical and Public Health Institute, Allschwil, Switzerland

2 University of Basel, Basel, Switzerland

3 University of Glasgow, Glasgow, United Kingdom

4 Public Health Laboratory–Ivo de Carneri, Chakechake, Pemba, United Republic of Tanzania

5 Ministry of Health, Zanzibar Town, Unguja, United Republic of Tanzania

*Correspondence: s.knopp@swisstph.ch

Short title: Behavior change communication for schistosomiasis elimination

Keywords Attitude; Behavior change communication; Control; Elimination; Intervention; Knowledge; Practices; Schistosomiasis; Tanzania

Abstract

Background Schistosomiasis is a parasitic disease that mostly affects school-age children in sub-Saharan Africa. Preventive chemotherapy is the mainstay of control. Other interventions, including behavior change communication (BCC), are recommended to reduce transmission and ultimately achieve elimination. We determined the effect of BCC interventions on schistosomiasis-related knowledge, attitudes, and practices (KAP) among children who were exposed to different BCC intervention frequencies and durations within the 4-year SchistoBreak project in Pemba, Tanzania.

Methods Annual surveys were carried out from 2020 to 2024 in 18 primary schools. Schistosomiasis-related KAP were assessed in randomly selected children attending grades 3-5. BCC interventions were implemented for one period (4 schools), two periods with no gap (3 schools), two periods with a 1-year gap (1 school), or never (10 schools). Linear and logistic mixed-models with random effect were applied to assess associations between BCC exposure categories as predictors and knowledge or attitude scores, or unsafe washing practices as the outcome variable in 2024.

Results: A total of 4196 children participated in the surveys. Knowledge and attitude improved with continuous or repeated exposure to BCC interventions. In 2024, the knowledge scores were significantly higher in children who received BCC intervention once (difference: 1.2, 95% confidence interval (CI): 0.6-1.9) or twice (difference: 2.1, 95% CI: 1.4-2.7), as compared to children who never received BCC interventions. Children who were exposed to BCC interventions twice (difference: 1.2, 95% CI: 0.8-1.6) had significantly higher attitude scores in 2024. Washing practices improved regardless of whether or not children were exposed to BCC. The washing platforms installed in intervention areas were known by up to half of the

children exposed to BCC interventions, but their use was considerably lower, varying between 7.5% and 43.1%.

Conclusions We demonstrated that BCC interventions were able to improve knowledge and attitudes in school children. Repeated BCC interventions might be required for sustainable long-term impact.

Trial registration ISRCTN, ISRCTN91431493. Registered 11 February. 2020, https://www.isrctn.com/ISRCTN91431493

Author summary

Urogenital schistosomiasis is a parasitic disease that affects mainly children in resource-constrained communities that have no access to improved water supply. On the Zanzibar islands in Tanzania, urogenital schistosomiasis is targeted for elimination. To support this goal, besides other interventions, behavior change communication (BCC) measures were implemented in the SchistoBreak project from 2020 to 2024. Eighteen primary schools in the study area received interactive BCC for varying periods. Overall, 4196 children participated in annual surveys to assess their schistosomiasis-related knowledge, attitude, and practices (KAP). Knowledge and attitude improved with continuous or repeated exposure to BCC interventions. In 2024, the knowledge and attitude scores were significantly higher in children who had received BCC intervention twice, as compared with children who never received BCC interventions. Washing practices improved, regardless of whether or not children were exposed to BCC. The safe washing platforms installed in intervention areas were known by up to half of the children exposed to BCC interventions, but their use was much lower (7.5-43.1%). Improved KAP is important to support schistosomiasis elimination through behavior change. For a lasting effect, repeated BCC interventions are necessary and they should be coupled with access to improved water infrastructure.

Background

Schistosomiasis is a neglected tropical disease caused by parasitic blood flukes of the genus *Schistosoma*. The disease mainly occurs in tropical and subtropical countries, with the highest disease burden concentrated in sub-Saharan Africa [1, 2]. Individuals of all age are susceptible to *Schistosoma* infection, but school-age children are at highest risk, explained by their behavior of frequently using infested freshwater bodies [3, 4]. Indeed, schistosomiasis transmission occurs at freshwater bodies where children play, bathe, or fetch water for domestic chores [5].

Preventive chemotherapy with praziquantel is the global strategy to control schistosomiasis morbidity [6]. Year after year, praziquantel is administered to millions of individuals in endemic countries, particularly to school-age children [7]. Preventive chemotherapy programs implemented over the past 20 years in sub-Saharan Africa have contributed to a substantial decrease in the prevalence and intensity of infection, and hence, morbidity due to schistosomiasis [8]. In addition to preventive chemotherapy, the World Health Organization (WHO) recommends environmental interventions, improved access to water, sanitation, and hygiene (WASH), and behavior change measures to reduce transmission in endemic settings [6]. Such an integrated intervention approach can also lead to interruption of transmission [9, 10]. Moreover, environmental and behavioral factors are considered essential to prevent the rebound of *Schistosoma* infections, alongside preventive chemotherapy programs and snail control measures [6].

Behavior change communication (BCC) is employed to deliver tailored messages concerning a particular subject to a target population, with the objective of initiating positive changes in behavior [11]. For example, BCC can be used to improve people’s health literacy [11]. Indeed, better disease-related knowledge can change attitudes, resulting in the adoption of sustainable

healthier behaviors and practices [12]. Since *Schistosoma* infection and transmission occur at freshwater bodies, BCC intervention strategies for schistosomiasis prevention are aimed at reducing or stopping people's water contact behaviors [13]. Hence, these strategies may include BCC for promoting the use of protective equipment (e.g., wearing boots), the adoption of ground games for children (e.g., tug of war, hopscotch, and jump rope), improving WASH, and enhanced uptake of treatment by at-risk communities [14-17].

On the Zanzibar islands, periodic preventive chemotherapy campaigns with praziquantel targeting school-age children have been implemented since the early 2000s [18-20]. As a result, the prevalence of *Schistosoma haematobium* and morbidity due to urogenital schistosomiasis were substantially reduced [20]. In 2010, efforts to eliminate urogenital schistosomiasis as a public health problem and to interrupt transmission were intensified [21]. In addition to preventive chemotherapy provided in schools and communities across the islands, snail control and BCC measures were implemented in selected schools and communities as part of specific research projects [21-23]. In the SchistoBreak project, conducted from 2020 to 2024, *S. haematobium* hotspot areas in the North of Pemba received a combination of preventive chemotherapy, snail control, and BCC interventions, while low-prevalence areas were subjected to a surveillance-response approach [23].

The purpose of this study was to determine the effect of BCC interventions on schistosomiasis-related knowledge, attitudes, and practices (KAP) of schoolchildren that were exposed to different frequencies and durations of BCC interventions within the 4-year SchistoBreak project. Moreover, we assessed a potential association of the BCC exposure frequency with KAP or *S. haematobium* infection, as determined in the final survey of the SchistoBreak project in 2024.

Methods

Ethics statement

Before the onset of the SchistoBreak project, the study protocol received a waiver from the ethics committee of Northwestern and Central Switzerland in 2019 (EKNZ; project-ID: Req-2019-00951). The study protocol was granted ethical approval by the Zanzibar Health Research Institute (ZAHRI) in December 2019 (ZAHREC/03/PR/December/2019/12), which was renewed annually. The final extension was approved in March 2023 (ZAHREC/04/AMEND/MARCH/2023/03). The study was prospectively registered at ISRCTN (https://www.isrctn.com/ISRCTN91431493).

The local researchers informed potential study participants about the purpose and procedures of the study. Upon recruitment, children invited to participate were provided with an information sheet and informed consent form (ICF) for their parents/legal guardians to read and sign, respectively. Only children who submitted a written ICF signed by their parents/legal guardians were included in the study. Additionally, children aged 12–17 years were invited to provide written signed assent for their participation.

Kiswahili was the main language used for communication during information meetings, informed consent procedures, questionnaire surveys, and implementation of BCC intervention activities. All documents used for local application in the study were translated from English into Kiswahili with the support of the research team and validated prior to use.

Each participant who tested positive for microhematuria and/or was infected with *S. haematobium* was informed about their diagnostic result and offered praziquantel (40 mg/kg oral dose) free of charge for treatment together with biscuits and juice (if treatment was given by our team) or porridge (if treatment was given during an MDA campaign).

Study area and population

The Zanzibar islands are a semi-autonomous part of the United Republic of Tanzania. The population is predominantly Muslim, and Kiswahili and English are the two official languages [24].

The SchistoBreak project was conducted on Pemba Island from 2020 to 2024 [23]. According to the national census of 2022, Pemba had a population of 543,500 inhabitants [24]~~. Pemba~~ is divided into four districts; namely, Chake Chake, Micheweni, Mkoani, and Wete, which are further subdivided into smaller administrative areas, known as shehias. ~~Th[illegible] SchistoBreak project~~ area consisted of 20 shehias in Wete and Micheweni districts. In 2020, 26 primary schools and 239 Islamic schools (madrassas) were located in the study area [25]. Children attend madrassas in addition to primary or secondary schooling [25-27]. ~~P[illegible]d~~ water ~~via taps~~ and wells ~~is~~ available to 54.5% and 34.5% of households, respectively [28]. For agricultural, domestic, minor industrial purposes, and some recreational activities, people rely mainly on groundwater such as rivers and streams, as tap water is often interrupted [28-30].

Study design

The SchistoBreak project followed a longitudinal ~~design~~ with four annual school-based and household-based surveys that used a cross-sectional sampling approach and three annual intervention periods [23]. ~~In~~ the surveys, the prevalence of *S. haematobium* infection was ~~assessed with~~ standardized, quality-controlled methods. ~~Besides parasitologic data, in the school-based surveys, data on children's schistosomiasis-related KAP were collecte[illegible]~~

Based on ~~the results of the parasitologic surveys~~, shehias were classified either as hotspot (*S. haematobium* prevalence $\geq 3\%$ in school children and/or $\geq 2\%$ in community members) or as low-prevalence areas (*S. haematobium* prevalence <3% in school children and <2% in

community members). ~~In the periods between the annual surveys, interventions for schistosomiasis elimination were implemented~~ (Fig 1). Hotspot shehias received a combination of at least annual preventive chemotherapy in schools and communities, snail control, and BCC interventions [23, 31, 32]. Low-prevalence areas were subjected to a targeted surveillance-response approach [23, 31]. This manuscript mainly focuses on the hotspot areas identified across the study period, including results about BCC interventions, schistosomiasis-related KAP, and *S. haematobium* infections in children attending grades 3-5.

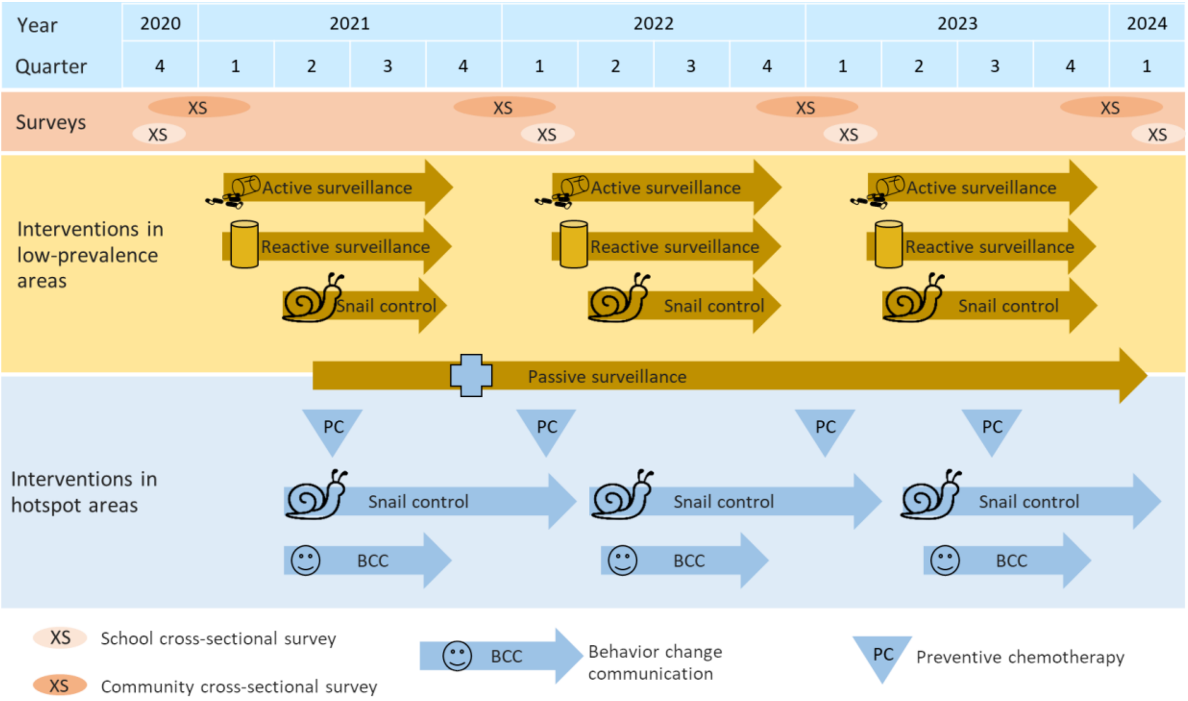

Fig 1. Parasitologic surveys and schistosomiasis control interventions for the SchistoBreak project in Pemba from 2020 to 2024. Parasitologic surveys and schistosomiasis control/elimination interventions in low-prevalence and hotspot areas of the SchistoBreak project in Pemba, Tanzania, from 2020 to 2024. Fig modified based on [23].

BCC interventions

The BCC interventions in our study consisted of three components. The first intervention component was community-based. It included the installation of two washing platforms per hotspot shehia and the organization of several community meetings. The washing platforms were constructed at a place that the study team had identified together with the community near an improved water source (e.g., well, pump, or tap) to provide a safe alternative to washing dishes or clothes at unimproved open freshwater bodies (e.g., rivers, streams, ponds, or lakes), where *S. haematobium* might be transmitted. The community meetings aimed to inform about the transmission of *S. haematobium*, symptoms of urogenital schistosomiasis, preventive strategies, and treatment.

The second intervention component was school-based. Teachers from public primary schools and madrassa were trained in classroom-based participatory teaching methods to educate children about schistosomiasis transmission and prevention using flip charts, snail boards, blood fluke pictures, life cycle drawing, and safe play methods [23, 33].

The third intervention component was also school-based and included health communication days called "Kichocho days" (note: kichocho is the Kiswahli term for schistosomiasis) [23, 33]. At Kichocho days, children watched and listened to dramas, songs, and games about schistosomiasis that their schoolmates performed in front of the whole school. Children learned about morbidity caused by urogenital schistosomiasis, the life cycle of *S. haematobium*, and measures to prevent an infection.

Cross-sectional school surveys

The annual school-based surveys were conducted in 18 public primary schools [25]. Children from nursery and grades 1-6 were eligible to participate in the parasitologic surveys, while

children attending grades 3-5 were additionally eligible to participate ~~in~~ the KAP surveys. If a school had more than one class per grade, ~~a~~ computer-generated randomization ~~list was~~ employed to select ~~either class A, B, C, or D~~, ~~respectively~~. ~~In each selecte[illegible]ass per grade, 25 children were randomly selected for participation,~~ using the following approach: all children in a class queued up, stratified by sex, and every third child in each line was systematically selected to be included in the study until 25 children were recruited [23]. Details of the sample size calculations are provided in the published study protocol [23]. The children were registered electronically and assigned a unique identifier (ID) code using tablet computers (Samsung Galaxy Tab A; Samsung Electronics, Seoul, South Korea, 2019) where Open Data Kit (ODK, www.opendatakit.org) software was installed.

After explaining the study procedures, children received an information sheet and ICF for their parents/legal guardians to sign. On the following day, the signed ICFs were collected. In addition, children aged 12 years and above were invited to sign their own assent forms. ~~Subsequently~~, children with signed ICF (and assent) were given a plastic container tagged with a QR code entailing their ID ~~and asked to provide~~ a fresh urine sample. ~~Children from grades 3-5 were also invited to participate in a questionnaire interview about schistosomiasis-related KAP~~. ~~For the interview, a study team member sat in private with each selected child and used a pretested ODK questionnaire [34]~~. ~~The questionnaire inquired about demographic data (e.g., age, sex, and shehia of residence of the child) and children's KAP regarding schistosomiasis transmission and prevention (e.g., knowledge of the lifecycle of *S. haematobium*, measures to prevent infection and avoid the spread of *S. haematobium*, and attitude toward schistosomiasis prevention and water contact practices).~~

Laboratory procedures

Urine samples were produced by the children between 10:00 and 14:00 hours in school. Samples were transferred to the Public Health Laboratory–Ivo de Carneri (PHL-IdC) in Chake Chake, Pemba, on the same day. In the laboratory, ~~urine samples were subjected to a filtration method~~. ~~For this purpose, each sample was~~ ~~mixed vigorously and 10 ml of urine was taken up with a syringe.~~ A filter holder (Millipore-Merck KGaA; Darmstadt, Germany) containing a 13 mm diameter fabric filter (Sefar Ltd.; Bury, United Kingdom) ~~was attached to the syringe and the urine was filtered~~. The filter was placed on a microscope slide, covered with hydrophilic cellophane soaked in glycerol, stained with Lugol's iodine and examined under a light microscope for *S. haematobium* eggs ~~by experienced laboratory technicians~~. The presence and number of *S. haematobium* eggs were recorded ~~on paper case report forms~~.

Data management

The registration and KAP questionnaire data were entered directly by the field enumerators into ODK using tablet computers and subsequently sent to a secure ODK server at the Swiss Tropical and Public Health Institute (Swiss TPH) in Allschwil, Switzerland. The laboratory data were recorded on paper case report forms by laboratory technicians. Data were double-entered into a Microsoft Excel database version 2016 (Microsoft Cooperation; Redmond Washington, United States of America) by two data entry clerks at the PHL-IdC and uploaded to a secure server at Swiss TPH. The data were checked for discrepancies using the statistical software R version4.4.0 (www.r-project.org). Errors were traced back to the original paper data forms and corrections were applied.

Statistical analysis

Data were analyzed using R version 4.4.0 (S1 Minimal dataset and S2 R-code). Only data from children attending grades 3-5 were included into the statistical analyses described here.

Schools participating in the study were stratified into BCC exposure categories: they were either not exposed to BCC at all, or exposed to BCC for one intervention period, for two intervention periods without a gap, or for two intervention periods with a 1-year gap.

Participants' sociodemographic data (i.e., sex, age, and school grade) were analyzed descriptively and presented as frequencies and percentages. Data from the KAP questionnaires were analyzed as follows: each of the three knowledge and two attitude multiple-choice questions were scored as incorrect (0 points), partially correct (0.5 points), and correct (1 point) with a cumulative highest possible score of 13 for knowledge and 9 for attitude, respectively, per annual survey. Subsequently, Jenks natural break was employed to classify the respective cumulative scores across all four surveys into distinct categories by minimizing variance within each category and maximizing variance between categories. Knowledge about schistosomiasis was classified into four categories: no knowledge (0 points), poor (1.0-1.5 points), moderate (1.5-2.5 points), and good (2.5-8.0 points) knowledge. Attitude was classified into three categories: poor (0-0.5 points), moderate (0.5-1.5 points), and good (1.5-5.5 points).

Practices for schistosomiasis prevention were analyzed descriptively from three questions about the main source of water children used for washing their bodies, dishes or clothes, ~~respectively.~~ Furthermore, data from the first and final surveys conducted at the start and end of the SchistoBreak project in 2020 and 2024, ~~respectively~~, were used to generate box plots, which illustrate the distribution of knowledge and attitude scores, ~~respectively~~, of children who were exposed to BCC for different intervention periods.

To assess a potential association of the number of intervention period categories (never, once, twice with no gap, and twice with a 1-year gap) with knowledge or attitudes of children, respectively, as categorical variables, a linear mixed-model with random effects was employed, adjusting for age and sex, and including the school as cluster variable. Of note, the categories for three schools that were exposed to BCC interventions twice with no gap and for the one school that was exposed to BCC interventions twice with a 1-year gap were combined to gain enough clusters for the analyses. The results were presented as estimated difference and 95% confidence intervals (CIs) using forest plots. Statistical significance was considered when the 95% CI of the difference did not include 0.

To assess a potential association of the number of intervention period categories (never, once, twice with no gap, and twice with a 1-year gap) with washing practices or *S. haematobium* infection, respectively, as binary variables, a logistic mixed-model with random effects was used, adjusting for age and sex, and including school as cluster variable. Washing practices were stratified into safe (if a child used either tap or well water for all washing practices) or unsafe (if a child used river/pond water for any washing practice). Participants were considered as *S. haematobium*-positive if at least one *S. haematobium* egg was detected in 10 ml of their urine. Of note, the categories for three schools that were exposed to BCC interventions twice with no gap and for the one school that was exposed to BCC interventions twice with a 1-year gap were combined to gain enough clusters for the analyses. The results were presented as odds ratio (OR) including 95% CIs using forest plots. Statistical significance was considered when the 95% CI did not include 1 for OR.

To ensure consistent handling of missing data (coded in R as “NA” [not available]) across the variables used in the models, conditional logic was applied. Hence, by default in R, the statistical analysis excluded all observations in variables containing missing data.

Results

Study participants

Over the whole SchistoBreak project, a total of 4626 children attending grades 3-5 in 18 schools were registered to participate in the study. Among them, 2852 children visited 10 schools that never received BCC interventions, 864 were from four schools that received BCC in one period, 632 were from three schools that received BCC interventions for two periods with no gap, and 278 were from the remaining school that received BCC interventions twice with a 1-year gap (Fig 2). Among the registered children, 79 did not participate in the KAP questionnaires.

From the 4547 students who participated in the KAP questionnaire surveys, the analyses included only those who visited a school that never received BCC interventions, or who participated in the surveys directly preceding or following a BCC intervention period. Hence, the KAP data from 2804 children who never received BCC, 632 children who received BCC once, 487 children who received BCC twice with no gap, and 273 children who received BCC twice with a 1-year gap were analyzed. Among those groups, 10, 8, 2, and 1 children, respectively, lacked urine filtration data.

In the 10 schools that never received the BCC interventions, 716 children participated in the KAP questionnaire survey in 2020, 660 in 2022, 725 in 2023, and 703 in 2024. In the four schools that received the BCC interventions for one period, 183 children were interviewed before and 218 children after the intervention. Additionally, 231 children were interviewed after the BCC interventions had stopped for 1 year. In the three schools that received the BCC interventions for two periods without a gap, 139 children were interviewed before the first, 161 children after the first and before the second intervention period, and 187 children after the

second intervention period. In the school that received the interventions twice with a 1-year gap, 66 children participated before the first BCC intervention, 65 after the first intervention, 71 after the 1-year gap, and 71 after the second intervention period.

Of the 4196 schoolchildren who were included in the statistical analyses, 2148 (51.2%) were females (Table 1). ~~Participants included 1414 (33.7%) children from grade 3, 1414 (33.7%) children from grade 4, and 1368 (32.6%) children from grade 5.~~ Thei~~r~~ interquartile age range~~d from~~ 10 to 12 years, with a median age of 11 years. Demographic details for each intervention exposure category group are summarized in Table 1.

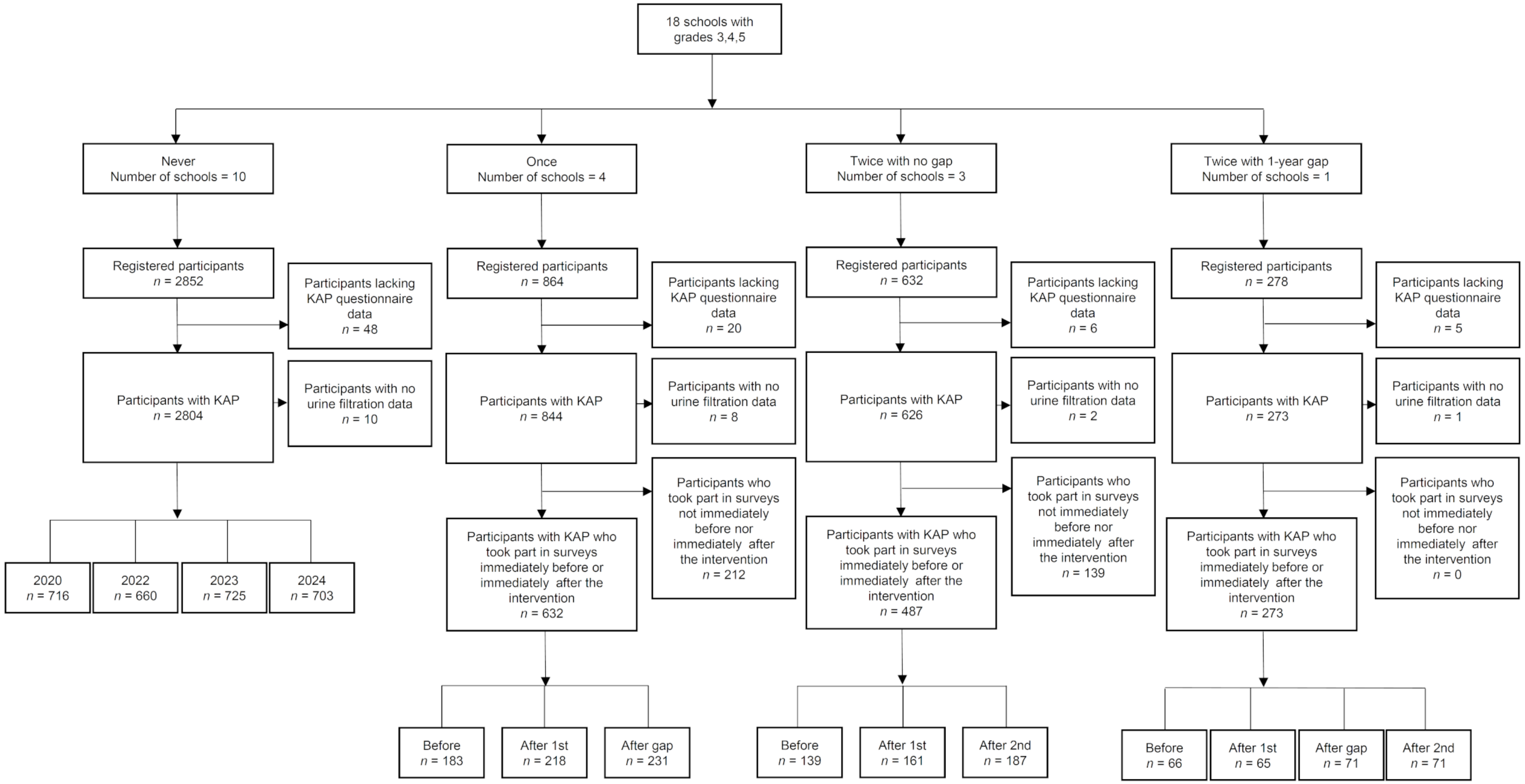

Fig 2. Exposure categories of schools to behavior change communication (BCC) interventions and participation in surveys from 2020-2024. Schools participation in behavior change communication (BCC) intervention periods and participation of children in questionnaire interviews about their schistosomiasis-related knowledge, attitude, and practices (KAP), and urine examinations for determining *Schistosoma haematobium* infection in Pemba, Tanzania, from 2020-2024.

Table 1. Characteristics of children participating in questionnaire interviews about their schistosomiasis-related knowledge, attitude, and practices (KAP) in 18 schools in Pemba, Tanzania, that were either not exposed to behavior change communication (BCC), or exposed to BCC for a different frequency of intervention periods, from 2020-2024. LIQR: lower interquartile range; UIQR: upper interquartile range.

		**Total** (*n* = 4196)		**Never received BCC interventions** (*n* = 2804)		**Received BCC interventions once** (*n* = 632)		**Received BCC interventions twice with no gap** (*n* = 487)		**Received BCC interventions twice with a 1-year gap** (*n* = 273)	
**Variable**	**Response**	***N***	**%**	***N***	**%**	***N***	**%**	***N***	**%**	***n***	**%**
**Sex**	Female	2148	51.2	1447	51.6	320	50.6	243	49.9	138	50.5
	Male	2048	48.8	1357	48.4	312	49.4	244	50.1	135	49.5
**Age (years)**	Median (LIQR - UIQR)	11 (10-12)		11 (10-12)		11 (10-12)		11 (10-12)		11 (10-12)	
**School grade**	3	1414	33.7	920	32.8	227	35.9	179	36.8	88	32.2
	4	1414	33.7	943	33.6	214	33.8	164	33.7	93	34.1
	5	1368	32.6	941	33.6	191	30.2	144	29.5	92	33.7

Knowledge of schistosomiasis prevention

In schools that never received BCC interventions, only a few children had good knowledge about schistosomiasis and around one-third had no knowledge in all four cross-sectional surveys (Fig 3A). In schools that received BCC interventions once, about one of eight children (13.1%) had good knowledge prior to the BCC interventions and one-fifth of the children (20.2%) had no knowledge (Fig 3B). After the interventions, the percentage of children with good knowledge increased (56.4%) and only few children had no knowledge (5.0%). After the interventions were ceased for 1 year, the percentage of children with good knowledge decreased (39.4%), while the percentage of children with no knowledge increased (13.4%).

In schools that received the BCC interventions twice without a gap, slightly less than one-fifth of the children (18.7%) had good knowledge and about one of eight children (12.9%) had no knowledge before the first BCC intervention period (Fig 3C). After the first intervention period, good knowledge increased (47.8%) and no knowledge decreased (3.7%). After the second intervention period, good knowledge increased further (65.2%) and the percentage of children with no knowledge remained low (6.4%).

In the school where BCC interventions were implemented twice with a 1-year gap, slightly less than one-fifth of the children (18.2%) had good knowledge, while the same percentage had no knowledge (18.2%) before the first BCC intervention (Fig 3D). After the first intervention period, good knowledge increased to about two-thirds (64.6%) and no knowledge decreased to very low levels (3.1%). After a 1-year gap of BCC interventions, the trend was somewhat reverted: the percentage of children with good knowledge decreased (29.6%) and the percentage of children with no knowledge increased (18.3%). Once the second period of BCC interventions was implemented, around two-thirds of the children had good knowledge (64.8%) and the percentage of children with no knowledge declined (4.2%).

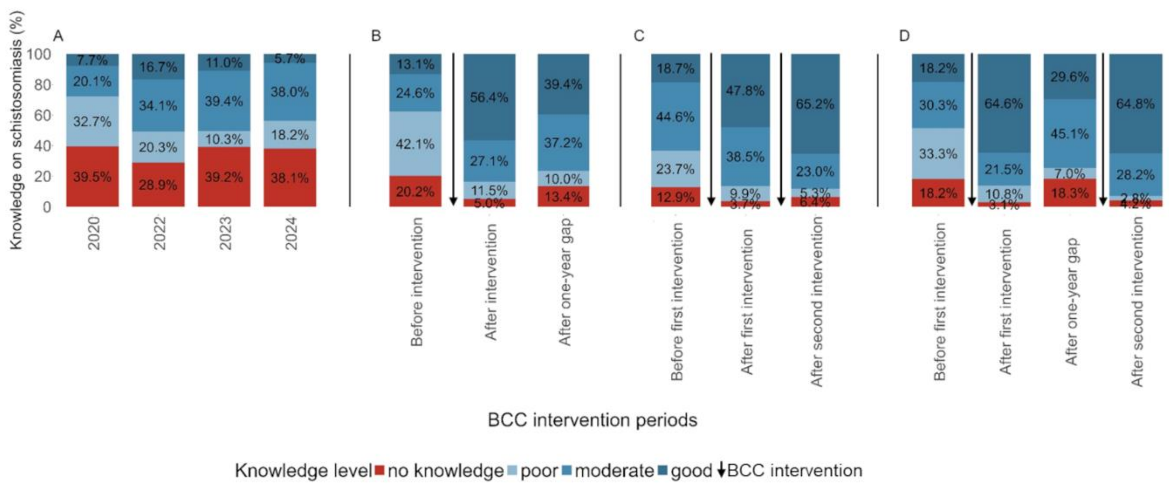

Fig 3. Schistosomiasis-related knowledge among children from 18 schools in Pemba, Tanzania. Schistosomiasis-related knowledge among children who were either not exposed to behavior change communication (BCC) (A; n = 10 schools), or exposed to BCC for one intervention period (B; n = 4 schools), for two intervention periods without a gap (C; n = 3 schools), or for two intervention periods with a 1-year gap (D; n = 1 school).

Attitude toward schistosomiasis prevention

~~In~~ schools that never ~~received~~ BCC interventions, only a ~~few~~ children ~~had~~ a good attitude about schistosomiasis prevention and transmission, whereas more than half of the children had a poor attitude in all four cross-sectional surveys (Fig 4A). In schools that received BCC interventions once, one-quarter of the children (25.1%) ~~had~~ a good attitude, ~~and~~ two-fifth of the children (39.9%) ~~had~~ a poor attitude prior to the BCC interventions (Fig 4B). ~~After~~ the interventions, the ~~percentage~~ of children with good attitudes increased (42.7%) ~~and~~ the percentage of children with poor attitudes decreased (23.4%). This trend was ~~reverted~~ ~~after stopping the BCC interventions and after 1 year~~, the ~~percentage~~ of children with a good attitude ~~decreased~~ ~~(~~25.5%~~)~~, ~~whereas the percentage of children with a poor attitude increased~~ ~~(~~34.2%~~).~~

In schools that received the BCC interventions twice without a gap, around one-fifth of the children (18.0%) had a good attitude, and two-fifths of the children (38.1%) had a poor attitude before the first BCC intervention period (Fig 4C). After the first intervention period, the percentage of children with a good attitude increased (24.8%), whereas poor attitude decreased (19.3%). This trend continued and after the second intervention period, almost half of the children had a good attitude (45.5%), while the percentage of children with poor attitudes slightly decreased (18.7%).

In the school where BCC interventions were implemented twice with a 1-year gap, about one-third of the children (30.3%) had a good attitude and one-third of the children (30.3%) had a poor attitude before the first BCC intervention period (Fig 4D). After the first intervention period, good attitude remained similar (32.3%) and poor attitude (15.4%) decreased. After a 1-year gap of no BCC interventions, the percentage of children with a good attitude decreased (21.1%) and the percentage of children with poor attitude increased (32.4%). Once the second period of BCC interventions was implemented, the trend was reverted, and more than half of the children had a good attitude (52.1%), while the percentage of children with a poor attitude decreased (16.9%).

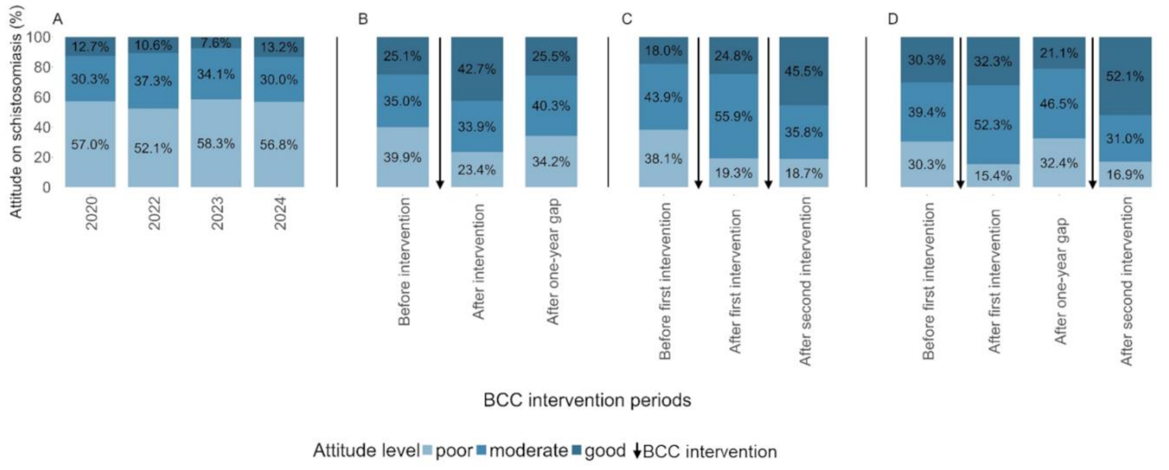

Fig 4. Schistosomiasis-related attitude among students from 18 schools in Pemba, Tanzania. Schistosomiasis-related attitude among students from 18 schools who were either not exposed to behavior change communication (BCC) (A; n = 10 schools), or exposed to BCC for one intervention period (B; n = 4 schools), for two intervention periods without a gap (C; n = 3 schools), or for two intervention periods with a 1-year gap (D; n = 1 school).

Use of water from open freshwater bodies for domestic activities

In schools that never received the BCC interventions, the percentage of children who reported using water from unimproved open freshwater bodies for washing clothes or dishes or were bathing decreased progressively from the first to the third cross-sectional survey and then increased in the final survey (Fig 5A).

In schools that received the BCC interventions once, the percentage of children who reported carrying out household chores at unimproved open freshwater bodies decreased for all activities after the BCC interventions (Fig 5B). After a 1-year gap of BCC interventions, the percentage of children who conducted their chores at ponds and rivers had increased.

In schools that received BCC interventions twice without a gap, the percentage of children who reported washing clothes or dishes and were bathing at unimproved open freshwater bodies

decreased progressively (Fig 5C). After the first intervention period, no child indicated the use of ponds or rivers for washing their body and after the second intervention period, no child reported using such freshwater bodies for washing dishes or their body.

In the school where BCC interventions were implemented twice with a 1-year gap, prior to the first BCC intervention, children only reported to use unimproved open freshwater bodies for washing clothes and their bodies (Fig 5D). After the first intervention, no child indicated to wash their body in ponds or rivers, and the percentage of children who used these locations for washing clothes declined. After a 1-year gap in BCC interventions, none of the children reported the use of water from unimproved sources for carrying out any of the domestic activities.

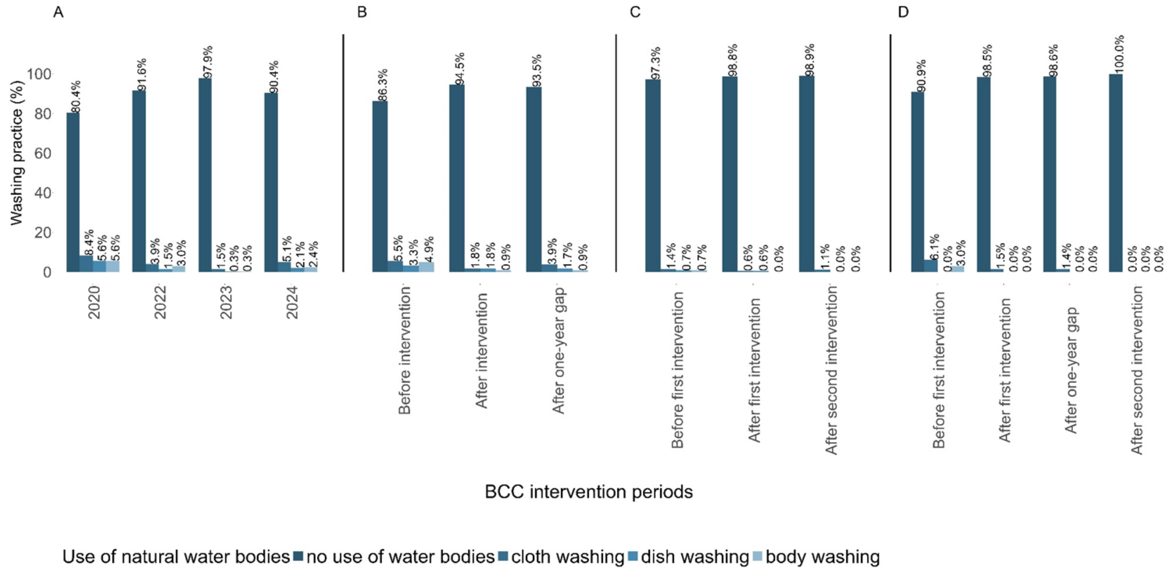

Fig 5. Water-related practices that exposed students from 18 schools in Pemba, Tanzania, to *Schistosoma haematobium* transmission. Students who were either not exposed to behavior change communication (BCC) (A; n = 10 schools), or exposed to BCC for one intervention period (B; n = 4 schools), for two intervention periods without a gap (C; n = 3 schools), or for two intervention periods with a 1-year gap (D; n = 1 school).

Awareness and use of washing platforms

Two washing platforms were installed in each hotspot shehia, in the year when the setting was characterized as a hotspot for the first time. In turn, shehias that were never considered a hotspot had no washing platforms installed.

In schools that never received the BCC interventions, the percentage of children who knew about and used washing platforms, respectively, was 1.1% and nil in 2020, 1.7% and 0.6% in 2022, nil and nil in 2023, and 0.3% and nil in 2024 (Table 2).

In schools that received BCC interventions once, before the first BCC intervention, only a few children knew (1.1%) about washing platforms but none of the children used them. After the first intervention period, the percentage of children who knew (24.3%) about the washing platforms and who used (12.4%) them increased. After the BCC interventions were ceased for 1 year, the percentage of children who knew (10.8%) and used (4.8 %) washing platforms had decreased.

In schools that received BCC interventions twice without a gap, only a small percentage of children knew (5.0%) about washing platforms before the intervention commenced, but no child used them. After the first intervention period, the percentage of children who knew (34.2%) and used (24.2%) the washing platform increased. After the second intervention period, the percentage of children who knew (25.1%) and used (7.5%) the washing platform decreased.

In schools that received the BCC interventions once with a 1-year gap, several children knew (12.1%) about washing platforms before the BCC interventions started, but no child used them. After the first BCC intervention period, more than half of the children knew (58.5%), but less than half used (43.1%) the washing platforms. After a 1-year gap, the percentage of children who knew (23.9%) and used (12.7%) the washing platforms decreased. After the second

intervention period, the percentage of children who knew (32.1%) about washing platforms increased, but the percentage of those who used (9.9%) the washing platforms decreased further.

Table 2. Awareness and use of washing platforms among children from 18 schools in Pemba, Tanzania, who were either not exposed to behavior change communication (BCC), or exposed to BCC for a different frequency of intervention periods, from 2020-2024.

	**Knowledge on washing platforms**		**Use of washing platforms**	
	**n/N**	**%**	**n/N**	**%**
**Never received BCC interventions**				
2020	8/716	1.1	0/726	0.0
2022	11/660	1.7	4/660	0.6
2023	0/725	0.0	0/725	0.0
2024	2/703	0.3	0/703	0.0
**Received BCC interventions once**				
Before intervention	2/183	1.1	0/183	0.0
After intervention	53/218	24.3	27/218	12.4
After one-year gap	25/231	10.8	11/231	4.8
**Received BCC interventions twice with no gap**				
Before first intervention	7/139	5.0	0/139	0.0
After first intervention	55/161	34.2	39/161	24.2
After second intervention	47/187	25.1	14/187	7.5
**Received BCC interventions twice with a 1-year gap**				
Before first intervention	8/66	12.1	0/66	0.0
After first intervention	38/65	58.5	28/65	43.1
After 1-year gap	17/71	23.9	9/71	12.7
After second intervention	23/71	32.4	7/71	9.9

Schistosomiasis-related knowledge and attitude in the final year of the SchistoBreak project

Figure 6A shows that in the final survey of the SchistoBreak project conducted in 2024, schistosomiasis-related knowledge in children who were exposed to BCC interventions was considerably better than in children who were never exposed to BCC interventions. Arithmetic mean and median knowledge scores increased with repeated and extended exposure to BCC interventions.

Figure 6B indicates that the arithmetic mean and median attitude scores were higher in children who were exposed to BCC interventions compared with those who were never exposed to BCC interventions. Arithmetic mean attitude scores increased with increasing exposure frequency and time.

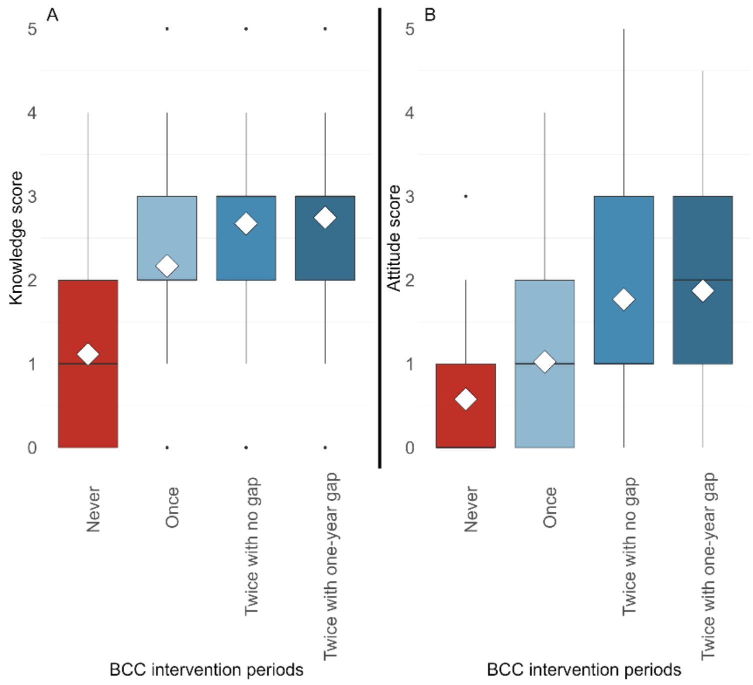

Figure 6. Distribution of knowledge scores (A) and attitude scores (B) of children from 18 schools in Pemba, Tanzania, who were either not exposed to behavior change communication (BCC) (n = 10 schools), or exposed to BCC for one intervention period (n = 4 schools), for

two intervention periods without a gap (n = 3 schools), or for two intervention periods with a 1-year gap (n = 1 school) in the final survey of the SchistoBreak project in 2024. The box plots show minimum, maximum, median, and interquartile ranges. The white diamond represents the arithmetic mean.

Association of BCC exposure with KAP and *S. haematobium* infection

In the final survey of the SchistoBreak project conducted in 2024, the knowledge scores about schistosomiasis were significantly higher in children who received the BCC intervention once (difference: 1.21, 95% CI: 0.58–1.85) or twice (difference: 2.08, 95% CI: 1.44–2.71), as compared with children w[illegible] never received BCC interventions. Boys had significantly higher knowledge scores (difference: 0.14, 95% CI: 0.00–0.27) than girls (Fig 7A). Increasing age had an inverse significant relationship (difference: -0.05, 95% CI: -0.10–0.00) with knowledge scores.

A similar trend was observed in attitude scores. Children who received BCC interventions once (difference: 0.47, 95% CI: 0.04–0.90) or twice (difference: 1.21, CI: 0.77–1.63) had significantly higher attitude scores compared with children who never received BCC interventions. Boys had significantly higher attitude scores (difference: 0.14, 95% CI: 0.03–0.24) than girls (Fig 7B). Increasing age had an inverse significant relationship (difference: -0.11, 95% CI: -0.15– -0.07) with attitude scores.

For practices on the use of unimproved water sources (Fig 7C) or for harboring a *S. haematobium* infection (Fig 7D), no differences in odds were observed between children who received BCC once or twice compared to children who never received BCC, and between boys and girls.

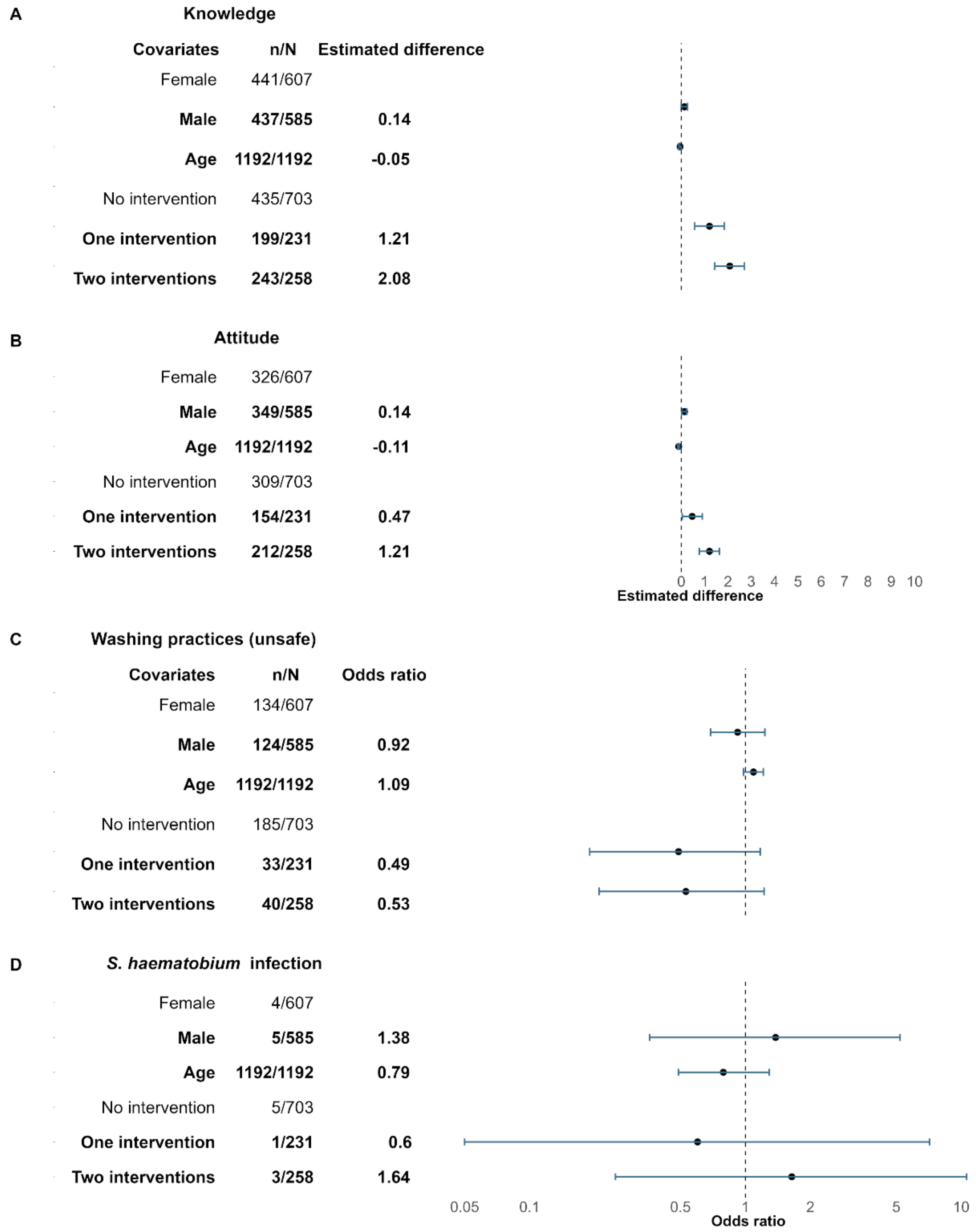

Fig 7. Association [illegible] behavior change communication (BCC) exposure frequency [illegible] knowledge score, attitude score, practices and *S. haematobium* infection. Association [illegible] behavior change communication (BCC) exposure frequency using a linear mixed-model for children's knowledge (A) and attitude (B), and using a logistic mixed-model for children's washing practices (C) and *S. haematobium* infection (D), as assessed in the final survey of the SchistoBreak project in Pemba, Tanzania, in 2024. Note that n/N represents the proportion of

children with knowledge, attitude, and practices (unsafe water use) and *S. haematobium* infection. The blue line shows the 95% confidence intervals (CIs). OR: odds ratio.

Discussion

Schistosomiasis primarily affects school-age children in sub-Saharan Africa [3, 4]. Preventive chemotherapy is the mainstay of the global schistosomiasis control strategy and has substantially reduced the prevalence and intensity of infection, ~~and~~ hence, morbi[illegible] [1]. Whenever resources allow, BCC measures are recommended to further [illegible] transmission, prevent the rebound of infections, and contribute to the ultimate goal of disease elimination [6, 9, 10]. We determined the effect of BCC interventions on schistosomiasis-related KAP [illegible] schoolchildren attending grades 3-5 who were exposed to BCC for a varying number of intervention periods during [illegible] 4-year SchistoBreak project implemented in Pemba, an area targeted for urogenital schistosomiasis elimination.

Our study showed that knowledge increased with repeated BCC intervention exposure. Moreover, children who received BCC interventions once or twice had considerably better schistosomiasis-related knowledge than those who never received BCC interventions at the end of the study. Good knowledge increased with repeated exposure. However, once BCC interventions were ~~term[illegible]ted~~ or interrupted for a certain period, the percentage of children with good knowledge decreased.

Interestingly, children from BCC intervention schools had more schistosomiasis-related knowledge preceding the interventions than children from non-intervention schools. Since the former schools were located in hotspot areas, where schistosomiasis has been a public health problem for a long time, children were probably more aware of the disease and its transmission than their counterparts from low-prevalence areas. Many children ~~[illegible] never received~~ BCC

interventions had no knowledge about schistosomiasis and the existing levels of knowledge remained relatively stable over time. These findings illustrate that knowledge acquired through BCC interventions is improved and accumulated over time and that long-term and repeated exposure to BCC is needed for a lasting effect. A positive impact of long-term BCC was also observed in Mozambique, where community members showed ~~an imp[illegible]ement in~~ knowledge about schistosomiasis after receiving a 1-year community dialogue intervention [35]. Moreover, studies from Brazil and a prior study in Zanzibar, where schistosomiasis-related BCC interventions were implemented for 2 and 4 years, respectively, showed that long-term exposure can have beneficial effects in creating awareness for preventive behaviors among children [13, 36]. Similar findings were reported in Ethiopia, where BCC interventions had a significant impact on improving knowledge and practices for other neglected tropical diseases, like soil-transmitted helminthiasis and onchocerciasis [12].

The BCC interventions applied in our study also had an obvious impact on the schistosomiasis-related attitude [illegible]children. ~~Indeed~~, children who received BCC interventions once or twice had significantly higher attitude scores than unexposed children in the final survey. Good and moderate attitude, which was reflected in knowledge about behaviors tha[illegible]~~n help not to get infected with~~ or transmit *S. haematobium* also increased over time with continuous or repeated implementation of BCC interventions. The attitudes that were assessed in our study were about behaviors that can prevent *S. haematobium* infection and transmission, including not urinating, playing, washing, or swimming in the river, but instead using clean water for washing, playing elsewhere, and taking treatment. Definitively, there is room for children to learn more and understand better what behaviors are needed to prevent infection and transmission. Future BCC interventions may need to emphasize these aspects ~~more forcefully~~. As discus[illegible] elsewhere and ~~rev[illegible]ed by our study~~, ~~le[illegible]ing and understanding need repeated exposure and take time, as does a change in behavior [37, 38]. Moreover, behavior change does not only need time but~~

~~also alternatives to the use of open freshwater bodies (e.g., access to improved WASH)~~ [34, 37, 39].

A substantial decline in the use of open freshwater bodies for washing clothes or dishes and bathing was found in children visiting schools of all investigated BCC exposure categories. Additionally, a profound decrease was ~~deter[illegible]ned~~ after the first intervention period and children who were exposed to BCC measures for two periods hardly report~~ed~~ the use of any natural open water bodies after the second intervention. However, a decrease was also detected in children from schools that were never exposed to BCC interventions, except [illegible] the final survey of our study. Hence, while children who were exposed to BCC interventions had likely adopted safer washing practices in line with what they learned, it may also be that the general water infrastructure in the North of Pemba was improved, as reported from other areas in Zanzibar [40].

In the hotspot shehias of our study area, but not in the low-prevalence areas, our study team together with the local communities, had installed two washing platforms near taps or wells as alternative options ~~to going~~ to ponds and rivers for washing clothes and dishes. Up to half of the children from hotspot schools that were exposed to BCC reported knowing about the washing platforms after the intervention period when platforms had been installed. The knowledge was partially lost when the BCC interventions were ceased or paused. The reported usage was low and related to BCC exposure in the preceding period and to the novelty of the platforms. These results point to the importance of keeping community members, including children, actively involved in the promotion of the use of safe alternatives, and in their maintenance, to ~~achieve long-term~~ sustainability of WASH infrastructure ~~that c[illegible] help to tackle~~ neglected tropical diseases like schistosomiasis [14, 17].

~~This study has specific strengths and limitations. We consider as strength and novelty that a~~ new BCC intervention approach was investigated in a 4-year study, allowing to assess ~~an~~ effect on schistosomiasis-related KAP across different periods of implementation. Important evidence was generated that documented the gain or loss of KAP with extended periods of intervention or when interventions are paused or stopped, respectively. Limitations of the study include the discrepancy in sample sizes in each intervention period, especially the very small number of participants in the category that received BCC intervention twice with a 1-year gap, which was not ideal for comparison. They also include the already existing schistosomiasis-related knowledge and attitude that children who attended hotspot schools had prior to the onset of our BCC interventions. Of note, after adjusting for the respective mean knowledge score and attitude score per school at baseline, the interpretation of our results in the final survey did not change. The random sampling approach used in the cross-sectional annual surveys did not allow the possibility to account for the change in knowledge and attitude scores for individual study participants between the surveys in 2020 and 2024. Hence, future studies may consider larger sample sizes in all comparison groups and assessing the effect of BCC in a “naïve” population.

Conclusion

In our study, knowledge and attitude about the life cycle of *S. haematobium*, places of transmission, ~~and~~ where and during what activities the parasite is transmitted increased with continuous or repeated exposure to BCC interventions, but was partially lost after 1-year gap in exposure or when interventions ceased altogether. R[illegible]rted washing practices like avoiding natural open freshwater bodies improved in children exposed and unexposed to BCC interventions. The few washing platforms installed in hotspot shehias were partially known but

not ~~muc[illegible]sed~~ by the study participants. For a maximum and lasting effect on KAP, BCC interventions should be applied over a long time and consolidated by improved access to WASH infrastructure in schools and communities. ~~Only when people know and are reminded repeatedly about the transmission route and consequences of schistosomiasis and when they have access to adequate alternatives, they may change their behavior to prevent infection and transmission, which will ultimately support elimination.~~

Acknowledgments

The authors wish to express their gratitude to all participating school children who participated in this study. We sincerely thank the teachers of the study schools and the team members at the Public Health Laboratory–Ivo de Carneri in Pemba, Tanzania, for their tremendous support in implementing the study activities.

Author contributions

Conceptualization of the study: NCN, LT, JH, and SK. *Data curation*: LT, NCN, and SK. *Formal analysis*: NCN and JH. *Funding acquisition*: SK and JU. *Investigation*: LT, UAM, MNA, SaMA, and SK. *Methodology*: NCN, LT, JH, and SK. *Project administration*: SaMA, and SK. *Resources*: ShMA, FK, JU, SaMA and SK. *Software*: NCN, LT, and JH. *Supervision*: LT, FK, JU, SaMA, and SK. *Visualization*: NCN, LT, JH, and SK. *Writing (original draft Preparation)*: NCN and SK. *Writing (review and editin*g): NCN, LT, JH, JU, and SK.

Availability of data and material

All relevant data are within the manuscript and its Supporting Information files.

Financial disclosure statement

This study obtained funding from the Swiss National Science Foundation (SNSF) via a PRIMA grant to Stefanie Knopp (PR00P3_179753). Naomi C. Ndum is supported by a personal stipend from the Swiss Government Excellence Scholarships (ESKAS) program and the Swiss Tropical and Public Health Institute (Swiss TPH). The funders had no role in study design, data collection and analysis, decision to publish, or preparation of the manuscript.

Competing interests

The authors have declared that no competing interests exist.

List of abbreviations

BCC	Behavior change communication
CI	Confidence interval
ICF	Informed consent form
ID	Identification
IQR	Inter quartile range
KAP	Knowledge, attitudes, and practices
ODK	Open Data Kit
OR	Odds ratio
PHL-IdC	Public Health Laboratory–Ivo de Carneri
WASH	Water, sanitation, and hygiene
WHO	World Health Organization

Supplementary Files

S1 Minimal Data Set and Dictionary (XLSX)

S2 Analysis Codes for "R" (R)

S3 STROBE checklist (PDF)

References

1. World Health Organization. Schistosomiasis and soiltransmitted helminthiases: progress report, 2022. Wkly epidemiol rec. 2023;98 [51]:667 - 76.

2. World Health Organization. Global report on neglected tropical diseases 2024. Geneva:World Health Organization:. 2024.

3. Gryseels B, Polman K, Clerinx J, Kestens L. Human schistosomiasis. Lancet. 2006;368:1106-18.

4. Colley DG, Bustinduy AL, Secor WE, King CH. Human schistosomiasis. Lancet. 2014;383:2253-64.

5. Osakunor DN, Woolhouse ME, Mutapi F. Paediatric schistosomiasis: What we know and what we need to know. PLoS Negl Trop Dis. 2018;12:e0006144.

6. World Health Organization. WHO guideline on control and elimination of human schistosomiasis. Geneva: World Health Organization; 2022.

7. Chanhanga N, Mindu T, Mogaka J, Chimbari M. The impact of targeted treatment and mass drug administration delivery strategies on the prevalence and intensity of schistosomiasis in school aged children in Africa: a systematic review. Infect Drug Resist. 2023:2453-66.

8. Kokaliaris C, Garba A, Matuska M, Bronzan RN, Colley DG, Dorkenoo AM, et al. Effect of preventive chemotherapy with praziquantel on schistosomiasis among school-aged children in sub-Saharan Africa: a spatiotemporal modelling study. Lancet Infect Dis. 2022;22:136-49.

9. Kajihara N, Hirayama K. The war against a regional disease in Japan. A history of the eradication of *Schistosomiasis japonica*. Trop Med Health. 2011;39:3.

10. Khieu V, Sayasone S, Muth S, Kirinoki M, Laymanivong S, Ohmae H, et al. Elimination of schistosomiasis mekongi from endemic areas in Cambodia and the Lao People's Democratic Republic: Current status and plans. Trop Med Infect Dis. 2019;4:30.

11. Ngigi S, Busolo DN. Behaviour change communication in health promotion: appropriate practices and promising approaches. Int J Innov Res Dev. 2018;7:84-93.

12. Abdissa D, Kebede Y, Morankar S, Abraham G, Bulcha G, Shiferaw T, et al. Effectiveness of integrated social and behavior change communication interventions in mass drug administration campaigns in enhancing knowledge, perceptions, and preventive practices for neglected tropical diseases in Jimma. Risk Manag Healthc Policy. 2024:2331-57.

13. Person B, Rollinson D, Ali SM, Mohammed UA, A'kadir FM, Kabole F, et al. Evaluation of a urogenital schistosomiasis behavioural intervention among students from rural schools in Unguja and Pemba islands, Zanzibar. Acta Trop. 2021;220:105960.

14. Torres-Vitolas CA, Trienekens SC, Zaadnoordijk W, Gouvras AN. Behaviour change interventions for the control and elimination of schistosomiasis: a systematic review of evidence from low- and middle-income countries. PLoS Negl Trop Dis. 2023;17:e0011315.

15. Kura K, Ayabina D, Hollingsworth TD, Anderson RM. Determining the optimal strategies to achieve elimination of transmission for *Schistosoma mansoni*. Parasit Vectors. 2022;15:55.

16. Pennance T, Person B, Muhsin MA, Khamis AN, Muhsin J, Khamis IS, et al. Urogenital schistosomiasis transmission on Unguja Island, Zanzibar: characterisation of persistent hot-spots. Parasit Vectors. 2016;9:1-13.

17. Ochola EA, Karanja DM, Elliott SJ. Local tips, global impact: community-driven measures as avenues of promoting inclusion in the control of neglected tropical diseases: a case study in Kenya. Infect Dis Poverty. 2022;11:81-90.

18. Stothard JR, French MD, Khamis IS, Basáñez M-G, Rollinson D. The epidemiology and control of urinary schistosomiasis and soil-transmitted helminthiasis in schoolchildren on Unguja Island, Zanzibar. Trans R Soc Trop Med Hyg. 2009;103:1031-44.

19. Guidi A, Andolina C, Makame Ame S, Albonico M, Cioli D, Juma Haji H. Praziquantel efficacy and long-term appraisal of schistosomiasis control in Pemba Island. Trop Med Int Health. 2010;15:614-8.

20. Trippler L, Knopp S, Welsche S, Webster BL, Stothard JR, Blair L, et al. The long road to schistosomiasis elimination in Zanzibar: a systematic review covering 100 years of research, interventions and control milestones. Adv Parasitol. 2023;122:71-191.

21. Knopp S, Mohammed KA, Ali SM, Khamis I, Ame SM, Albonico M, et al. Study and implementation of urogenital schistosomiasis elimination in Zanzibar [Unguja and Pemba islands] using an integrated multidisciplinary approach. BMC Public Health. 2012;12:1-13.

22. Knopp S, Person B, Ame SM, Ali SM, Hattendorf J, Juma S, et al. Evaluation of integrated interventions layered on mass drug administration for urogenital schistosomiasis elimination: a cluster-randomised trial. Lancet Glob Health. 2019;7:e1118-29.

23. Trippler L, Hattendorf J, Ali SM, Ame SM, Juma S, Kabole F, et al. Novel tools and strategies for breaking schistosomiasis transmission: study protocol for an intervention study. BMC Infect Dis. 2021;21:1024.

24. Office of Chief Government Statistician Z. The United Republic of Tanzania [URT], Ministry of Finance and Planning, Tanzania National Bureau of Statistics and President's Office - Finance and Planning, Office of the Chief Government Statistician, Zanzibar. The 2022 population and housing census: administrative units population distribution report; Tanzania, Zanzibar, December 2022. 2023.

25. Trippler L, Ali SM, Ame SM, Hattendorf J, Suleiman KR, Ali MN, et al. Fine-scale-mapping of *Schistosoma haematobium* infections at the school and community levels and intermediate host snail abundance in the north of Pemba Island: baseline cross-sectional survey findings before the onset of a 3-year intervention study. Parasit Vectors. 2022;15:292.

26. Celone M, Person B, Ali SM, Lyimo JH, Mohammed UA, Khamis AN, et al. Increasing the reach: involving local Muslim religious teachers in a behavioral intervention to eliminate urogenital schistosomiasis in Zanzibar. Acta Trop. 2016;163:142-8.

27. Pence A, Makokoro P, Ebrahim H, Barry O. Sankofa: Appreciating the past in planning the future of early childhood education, care and development in Africa. United Nations Educational, Scientific and Cultural Organization; 2023. p. 41.

28. Makame MO, Kangalawe RY. Water security and local people sensitivity to climate variability and change among coastal communities in Zanzibar. J Sustain Dev. 2018;11:23-32.

29. Mato R. Groundwater quality degradation due to salt water intrusion in Zanzibar Municipality. Afr J Environ Sci Technol. 2015;9:734-40.

30. Issa KM. Population growth and accessibility of water supplies in Tanzania: a case of urban west region–Zanzibar: The Open University of Tanzania; 2021.

31. Trippler L, Taylor L, Ali MN, Najim SO, Khamis KS, Hattendorf J, et al. Test-treat-track-test-treat [5T] approach for *Schistosoma haematobium* elimination on Pemba Island, Tanzania. BMC Infect Dis. 2024;24:1-16.

32. Trippler L, Ali SM, Masoud MO, Mohammed ZH, Amour AK, Suleiman KR, et al. Impact of chemical snail control on intermediate host snail populations for urogenital schistosomiasis elimination in Pemba, Tanzania: findings of a 3-year intervention study. Parasit Vectors. 2024;17:489.

33. Ndum NC, Trippler L, Mohammed UA, Ali AS, Hattendorf J, Utzinger J, et al. Capacities and needs of health care facilities for schistosomiasis diagnosis and management in elimination settings. Parasit Vectors. 2024;17:263.

34. Ndum NC, Trippler L, Najim SO, Ali AS, Hattendorf J, Ame SM, et al. One-year impact of behavioural interventions on schistosomiasis-related knowledge, attitude and practices of primary schoolchildren in Pemba, Tanzania. Infect Dis Poverty. 2024;13:84.

35. Rassi C, Martin S, Graham K, de Cola MA, Christiansen-Jucht C, Smith LE, et al. Knowledge, attitudes and practices with regard to schistosomiasis prevention and control: two cross-sectional household surveys before and after a community dialogue intervention in Nampula province, Mozambique. PLoS Negl Trop Dis. 2019;13:e0007138.

36. Murta FLG, Massara CL, Rodrigues MG, Beck LCNH, Favre TC. Teachers as multipliers of knowledge about schistosomiasis: a possible approach for health education programmes. BMC Infect Dis. 2022;22:853.

37. Kwasnicka D, Dombrowski SU, White M, Sniehotta F. Theoretical explanations for maintenance of behaviour change: a systematic review of behaviour theories. Health Psychol Rev. 2016;10:277-96.

38. World Health Organization. WASH and health working together: a" how-to" guide for neglected tropical disease programmes. Geneva: World Health Organization; 2023.

39. Wang S, Carlton EJ, Chen L, Liu Y, Spear RC. Evaluation of an educational intervention on villagers' knowledge, attitude and behaviour regarding transmission of *Schistosoma japonicum* in Sichuan province, China. Acta Trop. 2013;127:226-35.

40. Ministry of Health [MoH] [Tanzania Mainland], Ministry of Health [MoH] [Zanzibar], National Bureau of Statistics [NBS], Office of the Chief Government Statistician [OCGS]. Tanzania demographic and health survey and malaria indicator survey Dodoma, Tanzania, and Rockville, Maryland, USA: MoH, NBS, OCGS, and ICF; 2023.

Figure 1

Click here to access/download;Figure;Fig1_KAP2_19.03.2025.tif

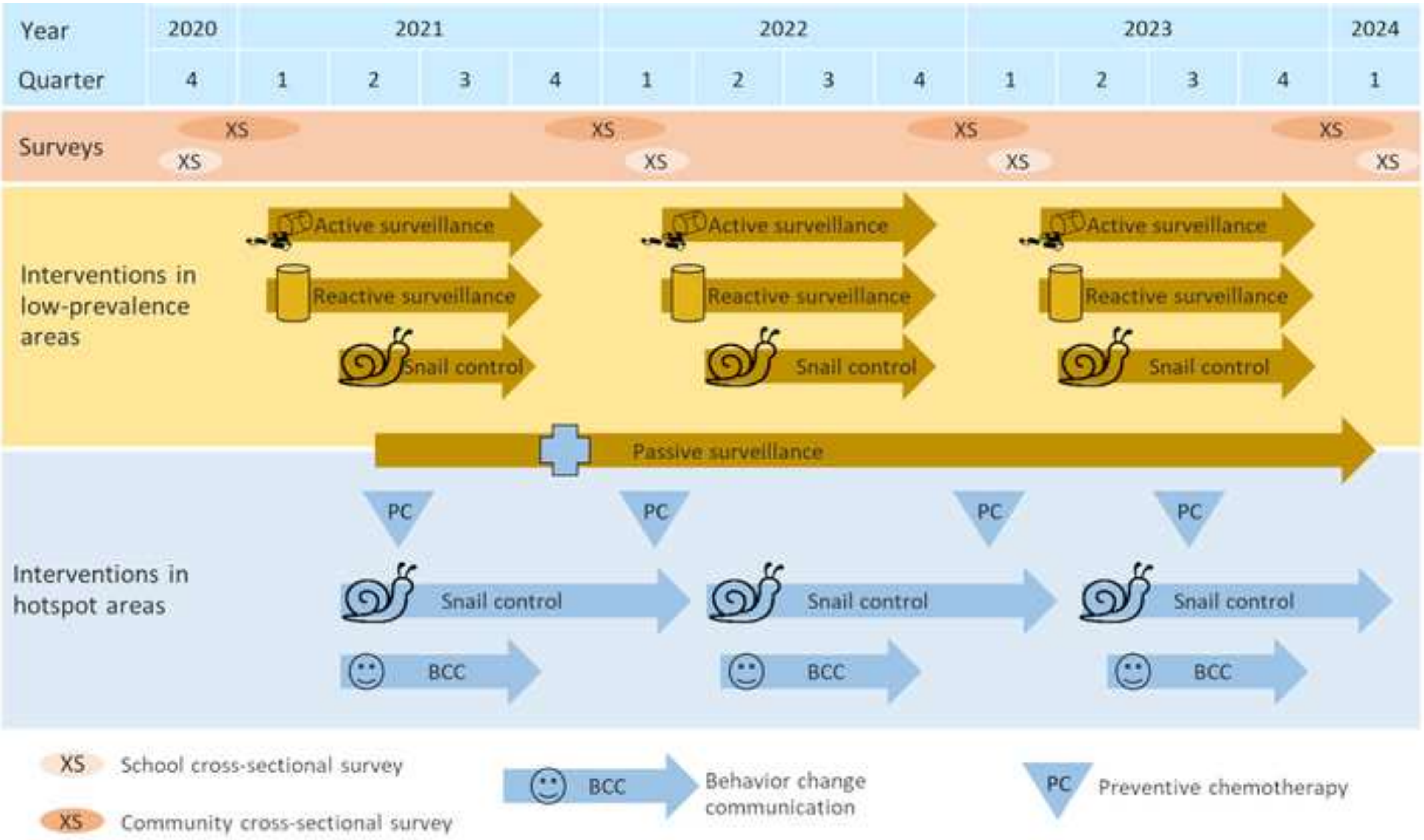

Figure 2

Click here to access/download;Figure;Fig2_KAP2_4.4.2025-large.tif

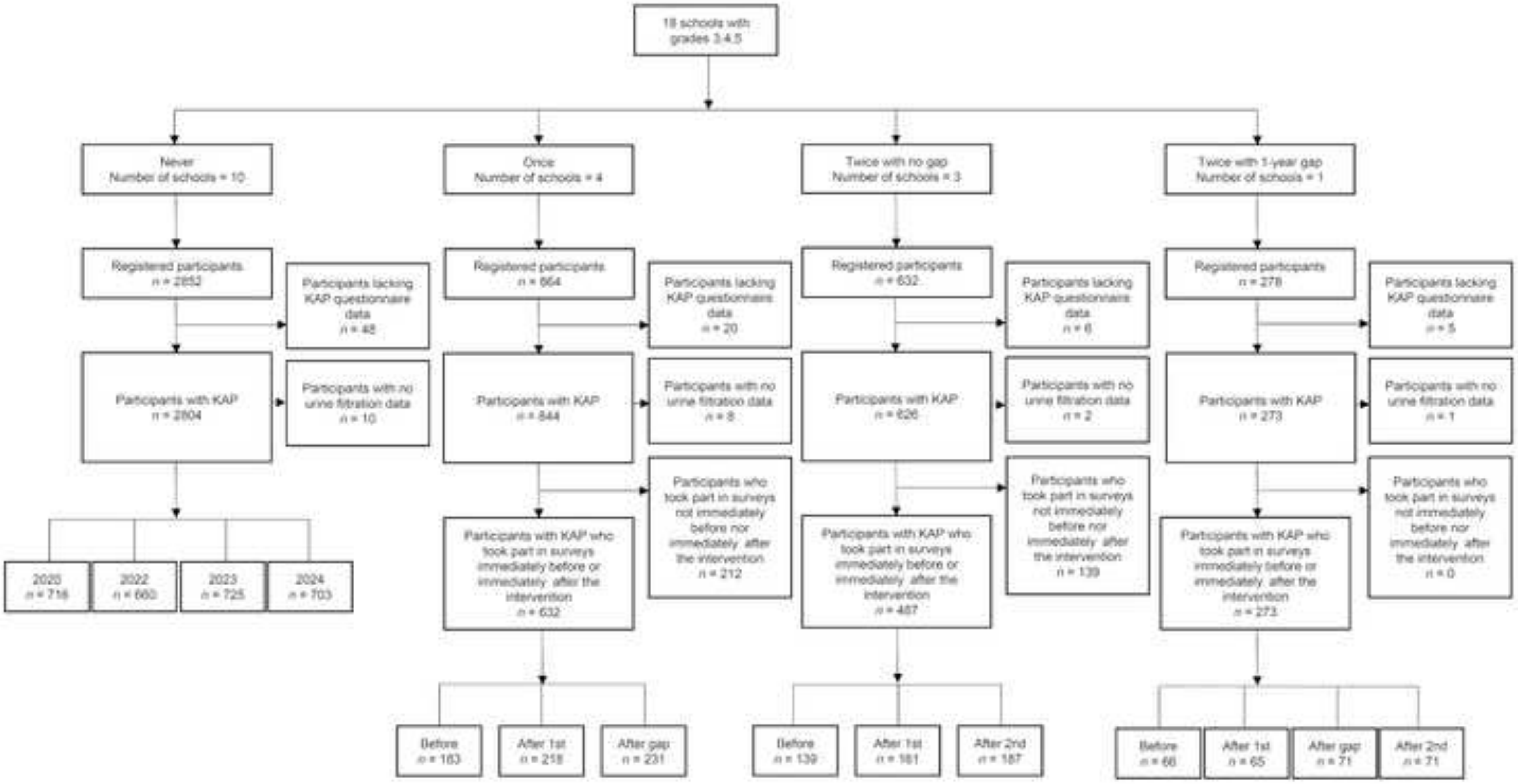

Figure 3

Click here to access/download;Figure;Fig3_KAP2_19.03.2025.tif

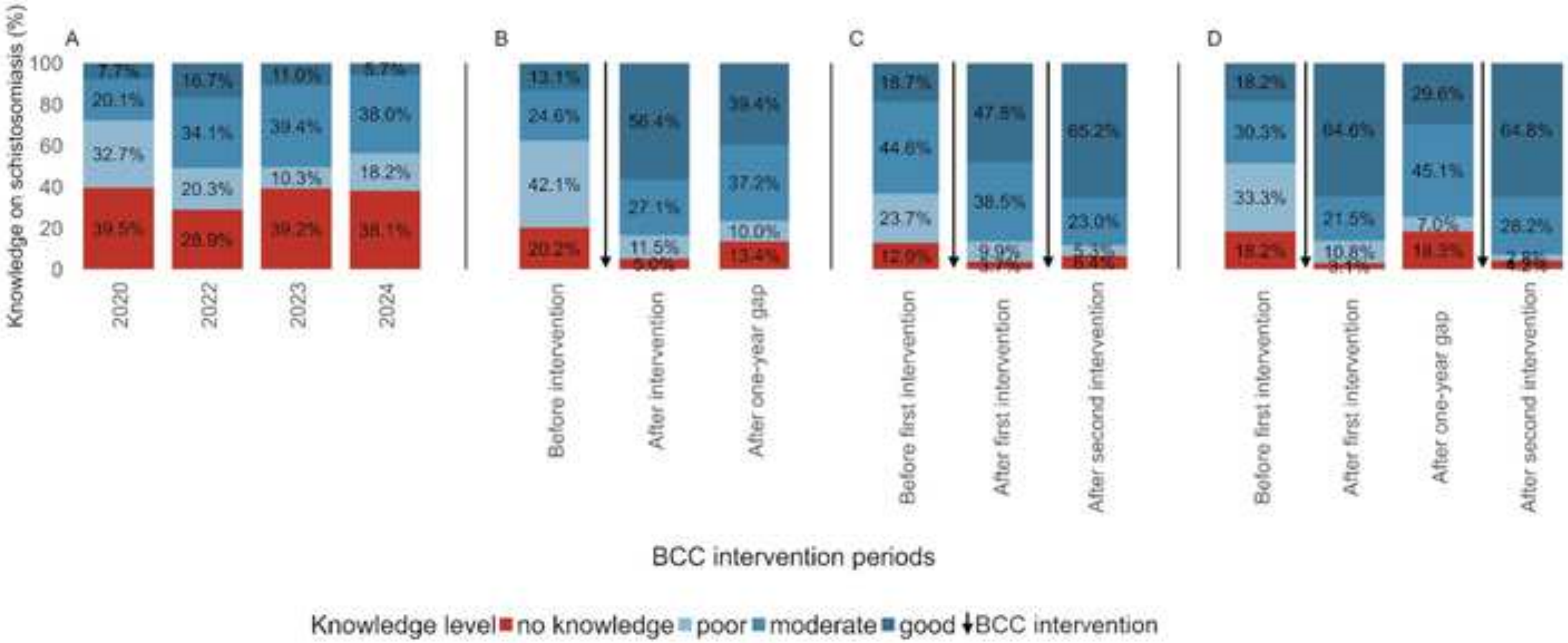

Figure 4 Click here to access/download;Figure;Fig4_KAP2_19.03.2025.tif

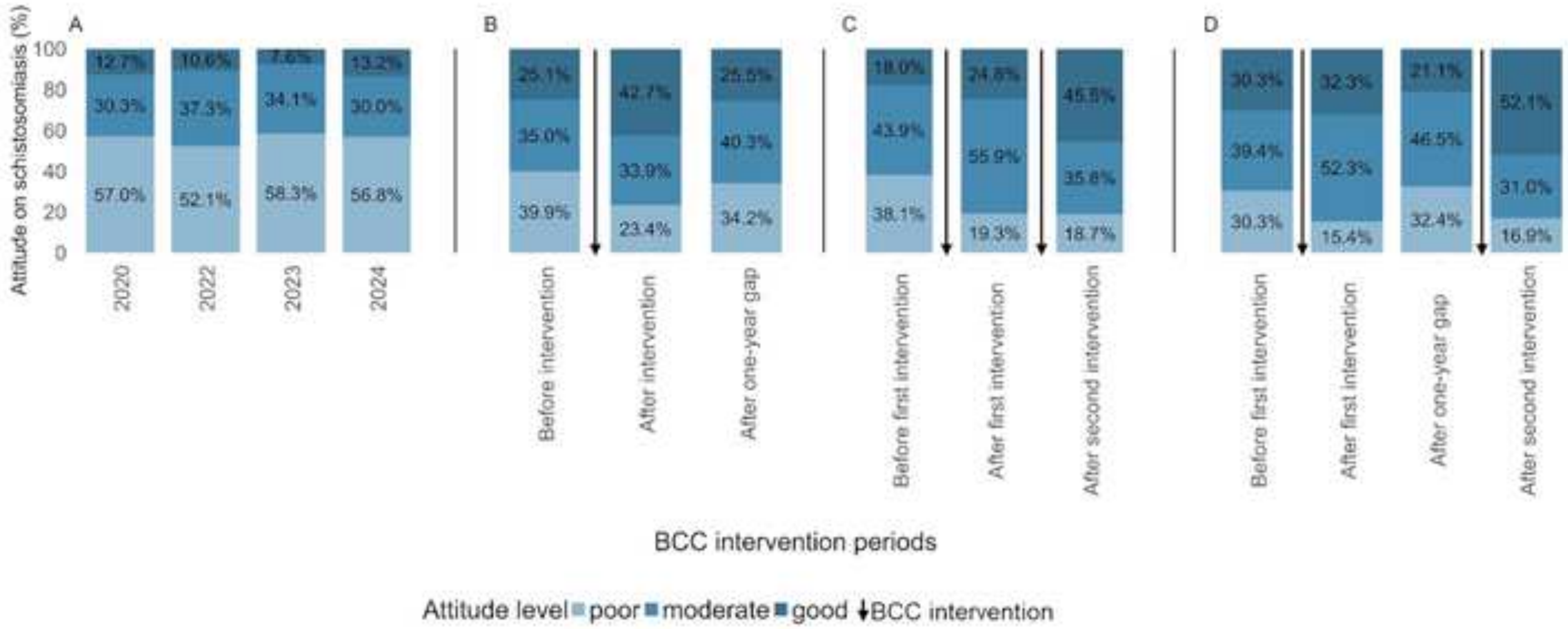

Figure 5

Click here to access/download;Figure;Fig5_KAP2_19.03.2025.tif

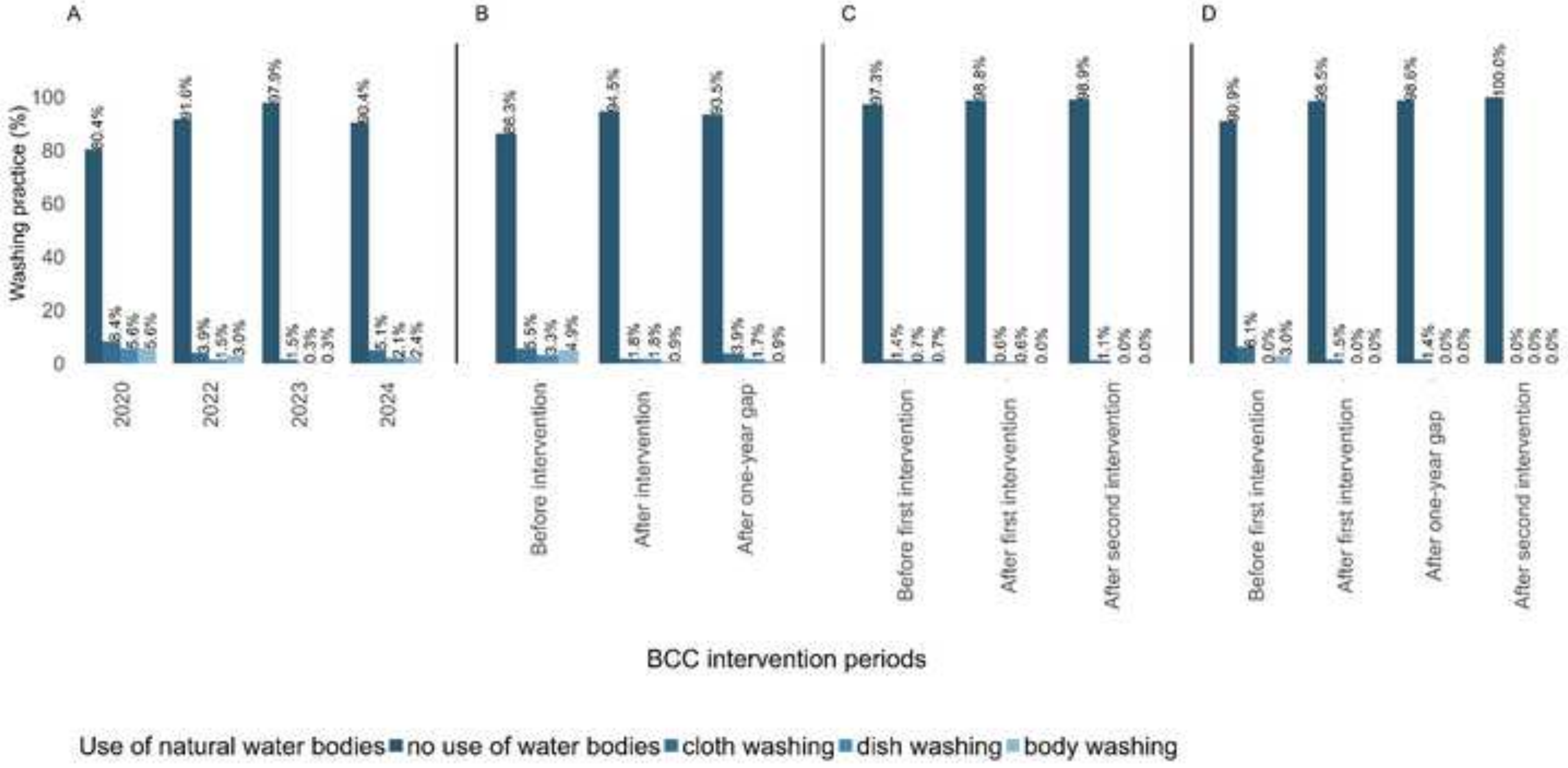

Figure 6

Click here to access/download;Figure;Fig6_KAP2_09.04.2025.tif

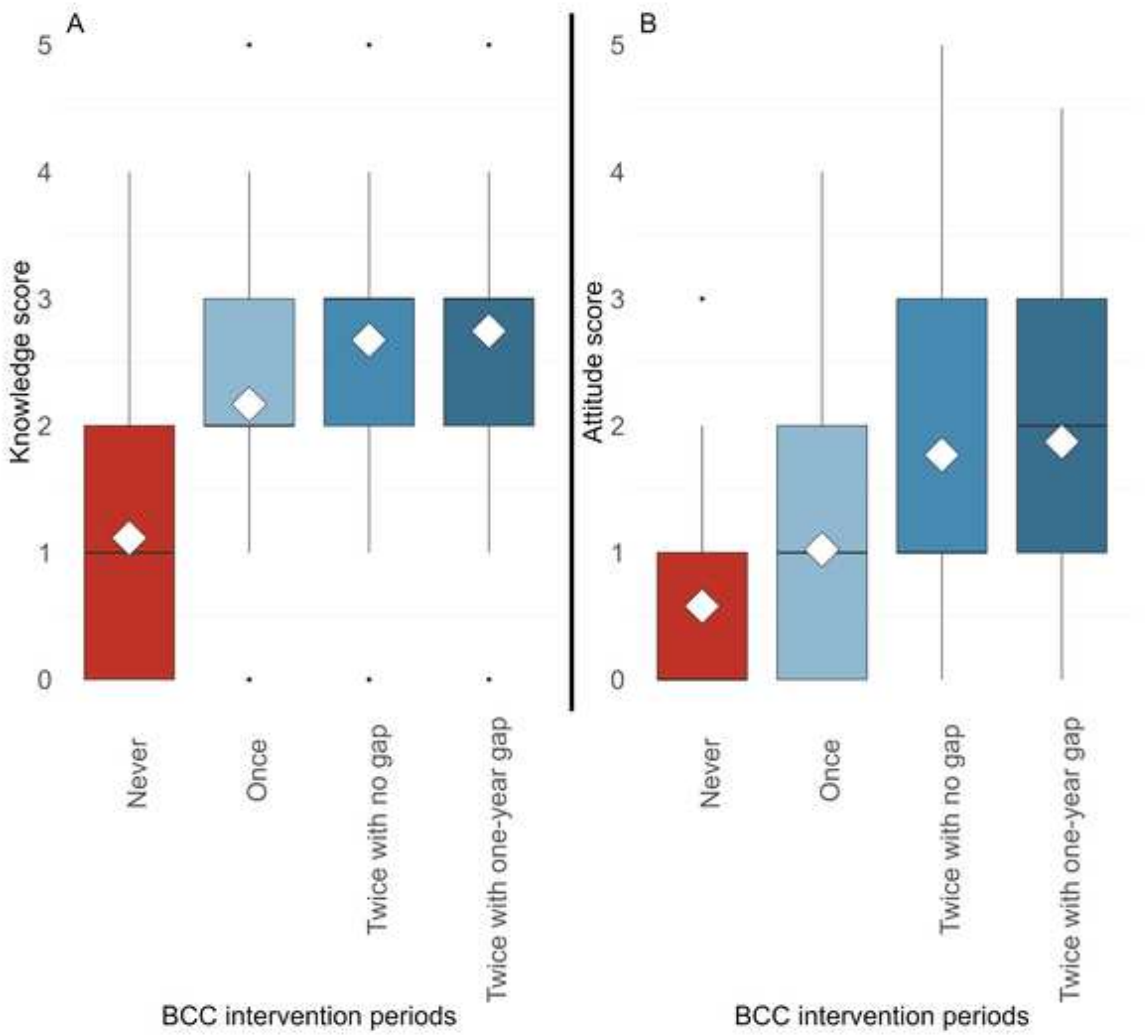

Figure 7

Click here to access/download;Figure;Fig7_KAP2_09.04.2025.tif

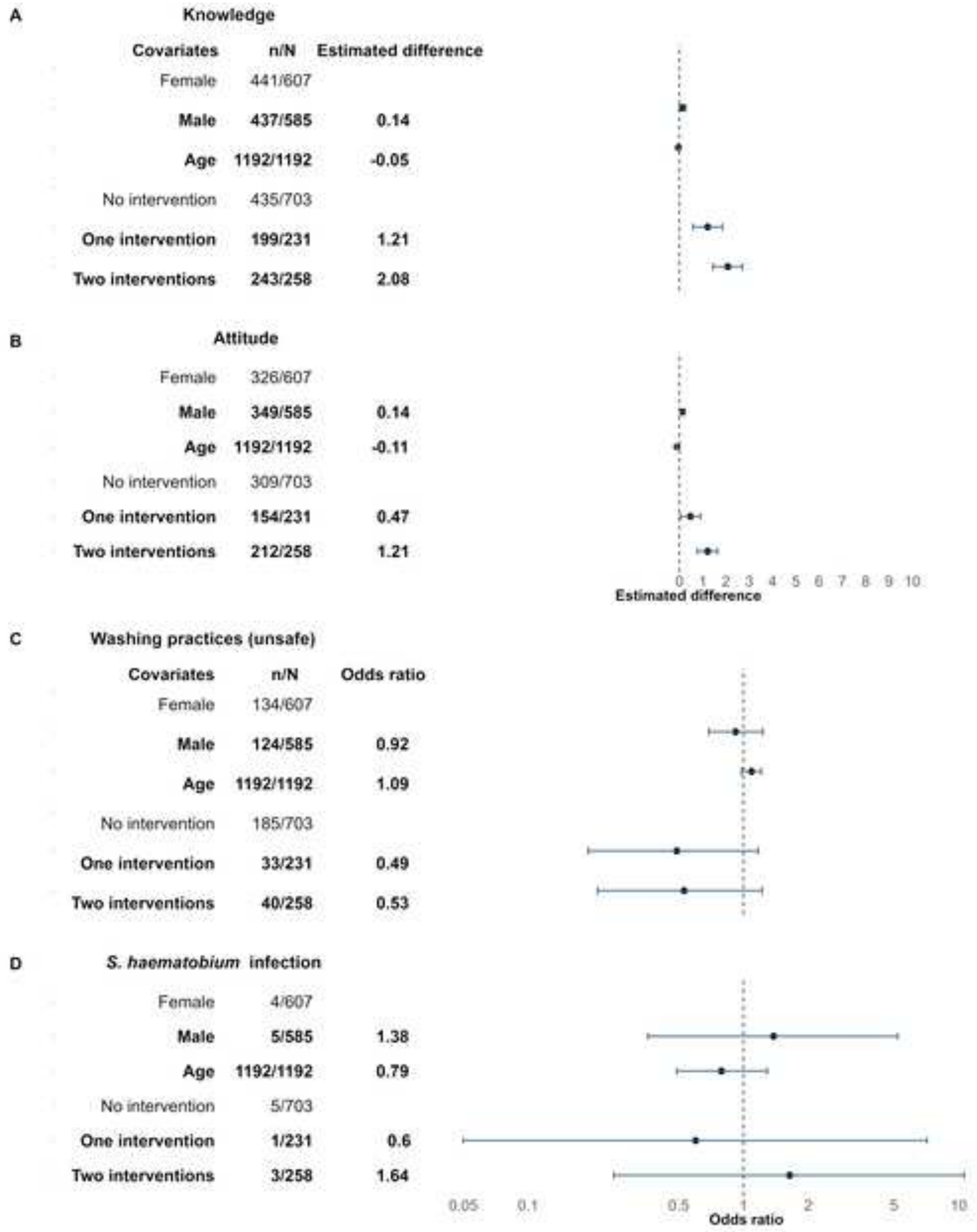

Click here to access/download
Supporting Information
S1_Minimal_Dataset_Dictionary.xlsx

Supporting Information 2

Click here to access/download
Supporting Information
S2_Analysis_Codes_R.R

Supporting Information 3

Click here to access/download
Supporting Information
S3_STROBE_checklist.pdf
