# Supplementary material for: Effect of behavioral interventions on schistosomiasis-related knowledge, attitudes, and practices of schoolchildren in Pemba, Tanzania: A 4-year repeated cross-sectional study
Source: PLoS Negl Trop Dis. 2025 Sep 30;19(9):e0013462. doi: 10.1371/journal.pntd.0013462 (PMC12483267; doi:10.1371/journal.pntd.0013462)
Supplement: Attachment — Submitted filename: PNTD-D-25-00558_Response_to_Reviewers_23.07.2025.pdf [file pntd.0013462.s008.pdf]

***Ndum et al.* "Effect of behavioral interventions on schistosomiasis-related knowledge, attitudes, and practices of school children in Pemba, Tanzania: a 4-year repeated cross-sectional study" (your reference no. PNTD-D-25-00558) – Point-by-Point response**

Allschwil, 23 July 2025

Dear Dr. Chippaux,

We were delighted to receive your e-mail dated 13 June 2025, which provided encouraging feedback from you and three external referees regarding the aforementioned manuscript. In the meantime, we carefully revised the manuscript in light of the reviewers' comments and suggestions and feel that the overall quality of our piece has been further improved.

Below, please find our point-by-point response, clearly indicating how and where in the manuscript changes have been made (line numbers). To assist you in readily tracking our amendments, we have used track-changes. Additionally, we submit a clean version of the revised manuscript.

We very much hope that this revised version is suitable for publication in *PLoS Neglected Tropical Diseases*. Hence, we look forward to your further disposition.

Yours sincerely,

Naomi Chi Ndum and Stefanie Knopp

(on behalf of all authors)

****

Dear Dr. Knopp,

Thank you for submitting your manuscript to PLOS Neglected Tropical Diseases. After careful consideration, we feel that it has merit but does not fully meet PLOS Neglected Tropical Diseases's publication criteria as it currently stands. Therefore, we invite you to submit a revised version of the manuscript that addresses the points raised during the review process.

We are delighted to read that you and the three reviewers feel that our manuscript has merit for publication. We have hence made an effort to revise our manuscript in light of the reviewers' comments and believe that the changes made have considerably improved the manuscript.

Please submit your revised manuscript within 60 days Aug 11 2025 11:59PM. If you will need more time than this to complete your revisions, please reply to this message or contact the journal office at plosntds@plos.org. When you're ready to submit your revision, log on to

https://www.editorialmanager.com/pntd/ and select the 'Submissions Needing Revision' folder to locate your manuscript file.

We have revised our manuscript in less than 60 days and resubmitted it on July 23, 2025.

Please include the following items when submitting your revised manuscript:

** A rebuttal letter that responds to each point raised by the editor and reviewer(s). You should upload this letter as a separate file labeled 'Response to Reviewers'. This file does not need to include responses to any formatting updates and technical items listed in the 'Journal Requirements' section below.*

We uploaded our point-by-point response to each comment raised by the editor and reviewers as "Response to Reviewers" via the online system.

** A marked-up copy of your manuscript that highlights changes made to the original version. You should upload this as a separate file labeled 'Revised Manuscript with Track Changes'.*

We uploaded a version indicating the changes made to the original version as "Revised Manuscript with Track Changes" via the online system.

** An unmarked version of your revised paper without tracked changes. You should upload this as a separate file labeled 'Manuscript'.*

Finally, we uploaded a clean version as "Manuscript" as a separate file via the online system.

If you would like to make changes to your financial disclosure, competing interests statement, or data availability statement, please make these updates within the submission form at the time of resubmission.

We adapted our financial disclosure, competing interests statement, and data availability statement within the submission form in line with the changes made in the manuscript.

Guidelines for resubmitting your figure files are available below the reviewer comments at the end of this letter.

Noted, thank you very much.

We look forward to receiving your revised manuscript.

Kind regards,

Jean-Philippe Chippaux, M.D., Ph.D.

Academic Editor

PLOS Neglected Tropical Diseases

Justin Remais

Section Editor

PLOS Neglected Tropical Diseases

Shaden Kamhawi

co-Editor-in-Chief
PLOS Neglected Tropical Diseases

orcid.org/0000-0003-4304-636XX

Paul Brindley

co-Editor-in-Chief

PLOS Neglected Tropical Diseases

orcid.org/0000-0003-1765-0002

Journal Requirements:

1) Some material included in your submission may be copyrighted. According to PLOSu2019s copyright policy, authors who use figures or other material (e.g., graphics, clipart, maps) from another author or copyright holder must demonstrate or obtain permission to publish this material under the Creative Commons Attribution 4.0 International (CC BY 4.0) License used by PLOS journals. Please closely review the details of PLOSu2019s copyright requirements here: PLOS Licenses and Copyright. If you need to request permissions from a copyright holder, you may use PLOS's Copyright Content Permission form.

Please respond directly to this email and provide any known details concerning your material's license terms and permissions required for reuse, even if you have not yet obtained copyright permissions or are unsure of your material's copyright compatibility. Once you have responded and addressed all other outstanding technical requirements, you may resubmit your manuscript within Editorial Manager.

We thank the journals' editorial team for flagging that some material included in our submission may be copyrighted. We have now created a new Figure 1 as described below and will hence not need any written permission from the copyright holder.

Potential Copyright Issues:

i) Figure 1. Please confirm whether you drew the images / clip-art within the figure panels by hand. If you did not draw the images, please provide (a) a link to the source of the images or icons and their license / terms of use; or (b) written permission from the copyright holder to publish the images or icons under our CC BY 4.0 license. Alternatively, you may replace the images with open source alternatives. See these open source resources you may use to replace images / clip-art:

- https://commons.wikimedia.org

- https://openclipart.org/.

We have revised Figure 1 and created all images/icons by hand using the graphic software Affinity Designer, Version 2.6.3. The background squares, timeline and the arrows and circles with written content were created by hand using Microsoft PowerPoint.

ii) Figure 1 appears to have been modified from a previously published figure. Please provide written permission from the copyright holder to publish this under our CC-BY 4.0 license, or remove the figure / replace the image. Please note we do not recommend using standard request forms available on Publishers' websites, as they grant single use rather than republication under an open access license.

We have redrawn Figure 1. Hence, we consider it a new figure. Of note, we removed the respective information and reference from the manuscript and figure caption (**see revised manuscript, lines 173−175**).

2) We note that your Data Availability Statement is currently as follows: "All relevant data are within the manuscript and its Supporting Information files.". Please confirm at this time whether or not your submission contains all raw data required to replicate the results of your study. Authors must share the "minimal data set" for their submission. PLOS defines the minimal data set to consist of the data required to replicate all study findings reported in the article, as well as related metadata and methods (https://journals.plos.org/plosone/s/data-availability#loc-minimal-data-set-definition).
For example, authors should submit the following data:

1) The values behind the means, standard deviations and other measures reported;

2) The values used to build graphs;

3) The points extracted from images for analysis.

Authors do not need to submit their entire data set if only a portion of the data was used in the reported study.

If your submission does not contain these data, please either upload them as Supporting Information files or deposit them to a stable, public repository and provide us with the relevant URLs, DOIs, or accession numbers. For a list of recommended repositories, please see [https://journals.plos.org/plosone/s/recommended-repositories.](https://journals.plos.org/plosone/s/recommended-repositories)

If there are ethical or legal restrictions on sharing a de-identified data set, please explain them in detail (e.g., data contain potentially sensitive information, data are owned by a third-party organization, etc.) and who has imposed them (e.g., an ethics committee). Please also provide contact information for a data access committee, ethics committee, or other institutional body to which data requests may be sent. If data are owned by a third party, please indicate how others may request data access.

We confirm that all relevant raw data required to replicate the results of our study are within the manuscript and its Supporting files. Specifically, we have shared in our submission the following files as Supplementary files (**see revised manuscript, lines 641−646**):

S1 Questionnaire (PDF)

S2 Minimal Data Set and Dictionary (XLSX)

S3 Analysis Codes for "R" (R)

S4 Table and Scoring System (PDF)

S5 STROBE checklist (PDF)

The "Minimal Data Set" contains the raw data used to generate our results on one sheet and a "Data Dictionary" to explain the variables and values in a second sheet. The "Analysis Code for R" provides the codes used to generate the means, standard deviations, graphs, and other measures reported in our manuscript. The Questionnaire, Table and Scoring System, Minimal Data Set and the R codes were cited within the text of the manuscript (**see revised manuscript, lines 242−244, lines 330−332, lines 362−365**).

3) Please amend your detailed Financial Disclosure statement. This is published with the article. It must therefore be completed in full sentences and contain the exact wording you wish to be published.

1) State what role the funders took in the study. If the funders had no role in your study, please state: "The funders had no role in study design, data collection and analysis, decision to publish, or preparation of the manuscript."

2) If any authors received a salary from any of your funders, please state which authors and which funders.

If you did not receive any funding for this study, please simply state: u201cThe authors received no specific funding for this work.u201d.

We have revised the Financial Disclosure statement as follows: “Funding for the study has been obtained from the Swiss National Science Foundation (SNSF; Bern, Switzerland) via a PRIMA grant (PR00P3_179753) of Stefanie Knopp. The salaries of Stefanie Knopp and Lydia Trippler were funded through this PRIMA grant. Moreover, Naomi C. Ndum is supported by a personal stipend from the Swiss Government Excellence Scholarships (ESKAS) program and the Swiss Tropical and Public Health Institute (Swiss TPH). The funders had no role in study design, data collection and analysis, decision to publish, or preparation of the manuscript.” **(see revised manuscript, lines 628−634).**

4) Please ensure that the funders and grant numbers match between the Financial Disclosure field and the Funding Information tab in your submission form. Note that the funders must be provided in the same order in both places as well.

We confirm that the funders and grant numbers match between the Financial Disclosure field and the Funding Information tab in our submission form.

Reviewers' Comments:

Reviewer's Responses to Questions

Key Review Criteria Required for Acceptance?

As you describe the new analyses required for acceptance, please consider the following:

Methods

-Are the objectives of the study clearly articulated with a clear testable hypothesis stated?

-Is the study design appropriate to address the stated objectives?

-Is the population clearly described and appropriate for the hypothesis being tested?

-Is the sample size sufficient to ensure adequate power to address the hypothesis being tested?

-Were correct statistical analysis used to support conclusions?

-Are there concerns about ethical or regulatory requirements being met?

Reviewer #1: YES

We thank Reviewer #1 for the thorough appraisal and feedback on our manuscript. We have addressed all the points he/she suggested below and feel that the overall quality of our manuscript has been further improved.

Reviewer #2: The methods used are appropriated. However, the following major concerns have emerged after review of the manuscript and will need to give more details/precisions:

1_The goal of this manuscript (Background) is unclear and will need to be addressed (Lines 104-111)

We thank Reviewer #2 for indicating that lines 104-111 of our background section were unclear. We have revised this section of the introduction, which provides background information about the schistosomiasis elimination efforts implemented in our study area, the Zanzibar islands, since 2010, and about the interventions that were implemented in the SchistoBreak project from 2020 to 2024 (**see revised manuscript, lines 101−111).**

The specific aim of the sub-study of the SchistoBreak project presented in our manuscript reads: "The study presented here focuses on the BCC interventions implemented within the 4-year SchistoBreak project across three intervention periods. Specifically, this study aimed to determine the effect of BCC interventions on schistosomiasis-related knowledge, attitudes, and practices (KAP) of schoolchildren that were exposed to different frequencies and durations of BCC interventions. Moreover, we assessed a potential association of the BCC exposure frequency with KAP or *S. haematobium* infection, as determined in the final survey of the SchistoBreak project in 2024." (**see revised manuscript, lines 112−118).**

2_ Some repetition on the manuscript. So you can delete some of them. For example:

We thank Reviewer #2 for several concrete suggestions of how to avoid repetition in our manuscript.

Lines 216-218: This has been summarily mentioned earlier in lines 128-133 under the ethical statement.

We agree that this part is repetitive and deleted the sentences.

Lines 220-222: (and asked provided…..): this has been mentioned in lines 206-207.

We agree and deleted the repetitive information.

Lines 271, 272, 273: Please delete "respectively"

We deleted "respectively", as suggested.

Reviewer #3: The article addresses schistosomiasis, a parasitic disease affecting school-aged children in sub-Saharan Africa, and evaluates the effectiveness of Behavior Change Communication (BCC) interventions as a complement to preventive chemotherapy.

The article is structured as follows:

Background

Methods

Results

Conclusions

This is a conventional and appropriate format for presenting public health research.

We thank Reviewer #3 for the valuable comments regarding the objective and structure of our manuscript.

Results

-Does the analysis presented match the analysis plan?

-Are the results clearly and completely presented?

-Are the figures (Tables, Images) of sufficient quality for clarity?

Reviewer #1: They are presented in an aggregated manner based on a score, which means that the detailed knowledge and behaviors that are so helpful for preparing, implementing and modifying BCC are missing. I suggest including the questionnaire as a supplementary file and adding a table showing the proportions of each type of knowledge or behavior over time.

We thank Reviewer #1 for his/her valuable suggestion and provide the questionnaire as well as a table showing the numbers of children who indicated respective (multiple choice) responses for of each type of knowledge or behavior question for the four exposure categories as supplementary information. We used numbers instead of proportions to account for the

possibility of multiple responses per question and to demonstrate that participants provided specific answers (**see revised manuscript, lines 214−216, lines 330−332 and lines 362−365 and “S1 Questionnaire. PDF” and “S4 Table and Scoring System. PDF”).**

Reviewer #2: Study participants is unclear and need to be written clearly. Please see some suggestions on the manuscript.

Idem with _Knowledge of schistosomiasis prevention and _ Attitude toward schistosomiasis prevention.

We thank Reviewer #2 for thoroughly scrutinizing our manuscript and for the suggestions provided in the annotated manuscript pdf. We have revised the manuscript accordingly, whenever we agree with the suggested changes.

Reviewer #3:

Setting and population: 18 primary schools in Pemba, Tanzania.

Timeframe: 2020 to 2024.

Sample: 4,196 children in grades 3 to 5, randomly selected.

Intervention groups:

One BCC intervention period (4 schools)

Two BCC periods consecutively (3 schools)

Two BCC periods with a 1-year gap (1 school)

No intervention (10 schools)

Statistical analysis: Linear and logistic mixed models with random effects to control for clustering.

Strengths of Methodology:

Longitudinal design over four years adds robustness.

Random selection of participants enhances generalizability.

Use of mixed models accounts for intra-school variability.

Potential Limitations:

Only one school in the "two periods with 1-year gap" group limits comparative power.

No qualitative data included to explore reasons behind behavioral outcomes.

We thank Reviewer #3 for the thorough appraisal of our methodology and for the feedback on its strengths and limitations. In this regard, we have amended the discussion as follows:

"A key strength and novel aspect is that the new BCC intervention approach was investigated in a 4-year study, allowing to assess its effect on schistosomiasis-related KAP across different periods of implementation. Important evidence was generated that documented the gain or loss of KAP with extended periods of intervention or when interventions were paused or stopped, respectively. The longitudinal design over four years adds robustness to the study outcomes. Moreover, the random selection of participants enhances generalizability of our findings, and the use of mixed models accounts for intra-school variability. Limitations of the study include the discrepancy in sample sizes in each intervention period. Especially the very small number of participants and that only one school was included in the category that received BCC intervention twice with a 1-year gap limits the comparative power." (see **revised manuscript, lines 573−583**).

With regard to the limitation that we did not include any qualitative data to explore reasons behind the behavioral outcomes, we are happy to let the reviewer know that we have a qualitative manuscript pertaining to a similar topic in preparation. In this manuscript we explore community perceptions about improvement and sustainability of behavior change communication interventions for schistosomiasis elimination.

Conclusions

-Are the conclusions supported by the data presented?

-Are the limitations of analysis clearly described?

-Do the authors discuss how these data can be helpful to advance our understanding of the topic under study?

-Is public health relevance addressed?

Reviewer #1: YES

We are happy that Reviewer #1 confirmed that our conclusion reads fine.

Reviewer #2: The conclusions are supported by the data presented but will need some adjustment.

We thank Reviewer #2 for several suggestions on how to improve the conclusion in the annotated pdf. We have amended our manuscript accordingly, whenever we agree with the suggested changes." (see **revised manuscript, lines 600−602 and lines 604−608**).

Reviewer #3: Strengths:

Demonstrates that education can positively affect knowledge and attitudes.

Reinforces the importance of repeated health education for sustained impact.

Uses reliable analytical models for complex school-level data.

Weaknesses:

Behavioral change (washing practices) was not strongly linked to BCC exposure — suggesting that knowledge alone is not sufficient for behavior change.

Lack of insight into barriers to behavior adoption, such as cultural norms or infrastructure issues.

We thank Reviewer #3 for this very nice summary of our conclusion. Of note, we have addressed the last point in our discussion as follows: "…future studies may consider larger sample sizes in all comparison groups and assessing the effect of BCC in a "naïve" population. Moreover, barriers to behavioral adoption such as cultural norms or infrastructural issues may be explored in more detail and using qualitative research methods." (see **revised manuscript, lines 589−593**).

Editorial and Data Presentation Modifications?

Use this section for editorial suggestions as well as relatively minor modifications of existing data that would enhance clarity. If the only modifications needed are minor and/or editorial, you may wish to recommend "Minor Revision" or "Accept".

Reviewer #1: NO

Reviewer #2: (No Response)

Reviewer #3: Recommendations:

Incorporate qualitative methods in future studies to explore why children may not act on their knowledge.

We thank Reviewer#3 for this recommendation. As indicated above, we have added this recommendation to the discussion as follows: "Moreover, barriers to behavioral adoption such as cultural norms or infrastructural issues may be explored in more detail and using qualitative research methods." (see **revised manuscript, lines 592−593**).

Combine BCC with structural interventions to improve water, sanitation, and hygiene (WASH) infrastructure.

We absolutely agree with this recommendation. In the conclusions it reads: "For a maximum and lasting effect on KAP, BCC interventions should be applied over a long time and consolidated by improved access to WASH infrastructure in schools and communities." (see **revised manuscript, lines 602−604**).

Tailor messages and tools to promote sustained behavioral change, not just awareness.

We thank Reviewer #3 for this suggestion. We absolutely agree that messages and tools should promote sustained behavioral change and are happy to let the reviewer know that we currently prepare a qualitative manuscript where we explore community perceptions about improvement and sustainability of behavior change communication interventions for schistosomiasis elimination.

Summary and General Comments

Use this section to provide overall comments, discuss strengths/weaknesses of the study, novelty, significance, general execution and scholarship. You may also include additional comments for the author, including concerns about dual publication, research ethics, or publication ethics. If requesting major revision, please articulate the new experiments that are needed.

Reviewer #1: General comment

The manuscript is related to the impact of behavior change interventions, which are an important schistosomiasis control strategy that is often neglected by control programmes. The article is well written, but some changes are required.

Specific comments
- Title: I suggest adding the type of study to the title: 'A four-year repeated cross-sectional study'.

We thank Reviewer #1 for this very valuable suggestion. We changed the title to: "Effect of behavioral interventions on schistosomiasis-related knowledge, attitudes, and practices of school children in Pemba, Tanzania: a 4-year repeated cross-sectional study." (see **revised manuscript, lines 1−2**).

- Keywords: Please add SAC.

We agree and added "School-age children" to the keywords (see **revised manuscript, lines 18−19**).

- Methods: Add a specific section on qualitative data collection and provide more details on the questions asked to the study participants.

We thank Reviewer #1 for raising this suggestion. Of note, in this study we did not collect any qualitative data. Using pre-tested questionnaires interviews, we collected data about children's schistosomiasis-related knowledge, attitude and practices (**see revised manuscript, lines 162−163**). The questionnaire with (multiple choice) questions can be found in our supplementary information (see **revised manuscript, lines 213−214, line 642 and "S1 Questionnaire.** PDF**")**.

- Results: They are presented in an aggregated manner based on a score, which means that the detailed knowledge and behaviors that are so helpful for preparing, implementing and modifying BCC are missing. I suggest including the questionnaire as a supplementary file and adding a table showing the proportions of each type of knowledge or behavior over time.

We thank Reviewer #1 for this valuable suggestion provide the questionnaire as well as a table showing the numbers for of each type of knowledge or behavior over time as supplementary information. We used numbers to account for the possibility of multiple responses per question and to demonstrate that participants provided specific answers (See **revised manuscript, lines 330−332, lines 362−365 and line 645 and "S1 Questionnaire. PDF" and "S2 Table. PDF"**).

Reviewer #2: This study describes and determines the effect of behavior change communication (BCC) interventions on schistosomiasis-related knowledge, attitudes, and practices (KAP) among children in different BCC intervention frequencies and durations in order to improve schistosomiasis elimination strategies. the experiences of implementing. Authors suggested some advices for BCC implementing strategies. This is certainly and interesting alternative social control method and will need to be published after revision.

We thank Reviewer #2 for his/her thorough appraisal of our manuscript and for recommending its publication after revisions are made.

Reviewer #3:

Conclusions and Policy Implications

BCC interventions are effective in improving knowledge and attitudes.

Repetition enhances the impact — one-off interventions may not be enough.

Behavior change lags behind knowledge and attitudes, indicating a need for:

Supportive environments (e.g., access to clean water and functional hygiene infrastructure).

Community-level engagement.

Monitoring actual usage, not just awareness.

We thank Reviewer #3 once more for his/her highly valuable input which has improved considerably the quality of our manuscript. His/her points above very nicely highlight the implications of our findings and we have incorporated some into our discussion and conclusion as follows:

"Finally, monitoring the actual use of WASH infrastructure and not just awareness will be important." (**see revised manuscript, lines 593−594**).

"In our study, knowledge and attitude about the life cycle of *S. haematobium*, places of transmission, and where and during what activities the parasite is transmitted increased with continuous or repeated exposure to BCC interventions but was partially lost after 1-year gap in exposure or when interventions ceased altogether. Reported washing practices were not strongly linked to BCC exposure suggesting that knowledge alone is not sufficient for behavior change. For a maximum and lasting effect on KAP, BCC interventions should be applied over a long time and consolidated by improved access to WASH infrastructure in schools and communities. Behavior change towards schistosomiasis prevention and altering transmission is more likely when individuals and the whole community are repeatedly informed about the disease transmission routes and consequences and engaged in taking up preventive behavior, and when they have access to adequate alternatives, which can ultimately support disease elimination efforts." (**see revised manuscript, lines 597−608**).

Figure resubmission:

While revising your submission, please upload your figure files to the Preflight Analysis and Conversion Engine (PACE) digital diagnostic tool, https://pacev2.apexcovantage.com/. PACE helps ensure that figures meet PLOS requirements. To use PACE, you must first register as a user. Registration is free. Then, login and navigate to the UPLOAD tab, where you will find detailed instructions on how to use the tool. If you encounter any issues or have any questions when using PACE, please email PLOS at figures@plos.org. Please note that Supporting Information files do not need this step. If there are other versions of figure files still present in your submission file inventory at resubmission, please replace them with the PACE-processed versions.

We have used PACE to ensure that all our figures meet PLOS requirements.

Reproducibility:

To enhance the reproducibility of your results, we recommend that authors of applicable studies deposit laboratory protocols in protocols.io, where a protocol can be assigned its own identifier (DOI) such that it can be cited independently in the future. Additionally, PLOS ONE offers an option to publish peer-reviewed clinical study protocols. Read more information on sharing protocols at https://plos.org/protocols?utm_medium=editorial-email&utm_source=authorletters&utm_campaign=protocols

We have no specific laboratory protocol, but our study protocol is published in: BMC Infect Dis. 2021 Sep 30;21(1):1024. doi: 10.1186/s12879-021-06620-8 **(please see revised manuscript, reference 23)**
